# Group-Based vs Individual Pelvic Floor Muscle Training to Treat Urinary Incontinence in Older Women: A Randomized Clinical Trial

**DOI:** 10.1001/jamainternmed.2020.2993

**Published:** 2020-08-03

**Authors:** Chantale Dumoulin, Mélanie Morin, Coraline Danieli, Licia Cacciari, Marie-Hélène Mayrand, Michel Tousignant, Michal Abrahamowicz

**Affiliations:** 1 School of Rehabilitation, Faculty of Medicine, Université de Montréal and Research Center of the Institut Universitaire de Gériatrie de Montréal, Montréal, Québec, Canada; 2 School of Rehabilitation, Faculty of Medicine and Health Sciences, Université de Sherbrooke, Research Center of the Centre Hospitalier de l’Université de Sherbrooke, Sherbrooke, Québec, Canada; 3 Research Institute of the McGill University Health Center, Department of Epidemiology, Biostatistics and Occupational Health, McGill University, Montreal, Québec, Canada; 4 Department of Obstetrics and Gynecology and Social and Preventive Medicine, Université de Montréal, Research Center of the Centre Hospitalier de l’Université de Montréal, Montréal, Québec, Canada; 5 Department of Epidemiology, Biostatistics, and Occupational Health, McGill University and Research Institute of the McGill University Health Center, Montreal, Québec, Canada

## Abstract

**Importance:**

Urinary incontinence is one of the most prevalent health concerns experienced by older women (aged ≥60 years). Individual pelvic floor muscle training (PFMT) is the recommended first-line treatment for stress or mixed urinary incontinence in women, but human and financial resources limit its delivery. Whether group-based PFMT performs as well as individual PFMT in this population remains unclear.

**Objective:**

To assess the efficacy of group-based PFMT relative to individual PFMT for urinary incontinence in older women.

**Design, Setting, and Participants:**

The Group Rehabilitation or Individual Physiotherapy (GROUP) study is a single-blind, randomized, noninferiority trial conducted in 2 Canadian research centers, from July 1, 2012, to June 2, 2018. A total of 362 community-dwelling women aged 60 years or older with symptoms of stress or mixed urinary incontinence were enrolled.

**Interventions:**

After an individual session conducted to learn how to contract pelvic floor muscles, participants completed 12-week PFMT as part of a group of 8 women (n = 178) or in individual sessions (n = 184).

**Main Outcomes and Measures:**

The primary outcome measure was the percentage reduction in urinary incontinence episodes at 1 year, as reported in a 7-day bladder diary and relative to pretreatment baseline. Secondary outcomes included lower urinary tract–related signs, symptoms, and quality of life immediately following treatment and at 1 year. Per-protocol analysis was used.

**Results:**

Among 362 women who were randomized (mean [SD] age, 67.9 [5.8] years), 319 women (88%) completed the 1-year follow-up and were included in the per-protocol analysis. Median percentage reduction in urinary incontinence episodes was 70% (95% CI, 44%-89%) in individual PFMT compared with 74% (95% CI, 46%-86%) in group-based PFMT. The upper boundary of the 95% CI for the difference in the percentage reduction in urinary incontinence episodes at 1 year was lower than the prespecified margin for noninferiority of 10% (difference, 4%; 95% CI, −10% to 7%; *P* = .58), confirming noninferiority. Individual PFMT and group-based PFMT had similar effectiveness for all secondary outcomes at 1 year. Adverse events were minor and uncommon.

**Conclusions and Relevance:**

Results of the GROUP study suggest that group-based PFMT is not inferior to the recommended individual PFMT for the treatment of stress and mixed urinary incontinence in older women. Widespread use in clinical practice may help increase continence-care affordability and treatment availability.

**Trial Registration:**

ClinicalTrials.gov Identifier: NCT02039830

## Key Points

**Question:**

Is pelvic floor muscle training (PFMT) delivered in a group setting noninferior to the recommended individual PFMT for urinary incontinence in older women (aged ≥60 years)?

**Findings:**

In this noninferiority randomized clinical trial of 362 older women with urinary incontinence, the median percentage reduction in incontinence episodes at 1 year was 70% in individual compared with 74% in group-based PFMT intervention. The difference between groups fell below the noninferiority margin of 10%, supporting noninferiority of group-based PFMT.

**Meaning:**

The findings of this trial show that group-based PFMT is noninferior to the recommended individual PFMT; widespread use in clinical practice could increase urinary incontinence treatment capacity for older women.

## Teaser

This randomized clinical trial demonstrates that outcomes of women participating in group-based pelvic floor muscle training are not inferior to outcomes of women receiving individualized training.

## Introduction

Urinary incontinence is one of the most prevalent health concerns confronting older women (aged ≥60 years).^1,2^ A medical and social problem, urinary incontinence shame and negative self-perception may lead to reduced social interaction and physical activity, interfering with healthy aging.^3,4,5,6^ Pelvic floor muscle training (PFMT) has been reported to be effective to cure or improve urinary incontinence symptoms in young, middle-aged, and older women with stress or mixed urinary incontinence.^7^ National and international clinical practice guidelines recommend supervised individual PFMT as first-line treatment for women with stress or mixed urinary incontinence.^8,9^ However, inadequate human and financial resources limit the delivery of PFMT.^10^ Consequently, and frequently, surgery is used as first-line therapy despite serious adverse effects,^5,11^ adding costs and pressure to the health care system. Pelvic floor muscle training delivered to a group of women rather than individually could overcome financial and human resource barriers.^12^ Group interventions are considered to be useful tools to promote behavior modification in the health promotion field, fostering peer support and discussion while increasing motivation by reducing stigma and isolation.^12^ Addressing urinary incontinence through group sessions could prove to be an effective means of encouraging active self-management, which is needed for long-term benefits. However, it is unclear whether group-based PFMT performs at least as well as the standard of care (individual PFMT), based on limited evidence.^8^

We conducted the Group Rehabilitation or Individual Physiotherapy (GROUP) study to determine whether the effectiveness of group-based PFMT is not inferior to individual PFMT in women aged 60 years or older with stress or mixed urinary incontinence.

## Methods

### Design

The GROUP study was designed as a single-blind, randomized, multicenter, noninferiority trial, with the primary objective of evaluating whether group-based PFMT was not inferior to individual PFMT for percentage reduction in urinary incontinence episodes 1-year postrandomization. Secondary objectives compared the 2 interventions immediately following treatment and at 1 year for the following variables: lower urinary tract–related signs, symptoms, and quality of life (QoL); urinary incontinence and PFMT self-efficacy; impression of improvement; satisfaction with treatment; and adverse events. Details of the study design were previously published^13^ and the study protocol is available in Supplement 1. The study protocol was approved by the research ethics boards at both research centers of the Institut Universitaire de Gériatrie de Montréal and the Centre Hospitalier Universitaire de Sherbrooke. The GROUP trial was conducted as per the original protocol, with adjustments explained in the Statistical Analysis section. Participants provided written informed consent; participants did not receive financial compensation.

The study was conducted from July 1, 2012, to June 2, 2018. Women were recruited from August 1, 2012, to May 1, 2017, through advertisements in community centers, newspapers, web and social media platforms, public conferences, a research bank of participants, and gynecology and urology clinics in the metropolitan areas of the 2 study centers (Montreal and Sherbrooke, Canada). Women were prescreened by telephone, and those meeting the inclusion criteria underwent subsequent on-site screening. Eligible participants were women aged 60 years or older with symptoms of stress or mixed urinary incontinence who reported at least 3 episodes of involuntary urine loss per week during the preceding 3 months.^14^ Stress and mixed urinary incontinence were confirmed using the validated Questionnaire for Incontinence Diagnosis.^15^ Exclusion criteria were body mass index (BMI) 35 or greater (calculated as weight in kilograms divided by height in meters squared), reduced mobility (requiring a mobility aid), chronic constipation,^16^ important pelvic organ prolapse (Pelvic Organ Prolapse Quantification System >stage 2),^17^ physiotherapy treatment or surgery for urinary incontinence or pelvic organ prolapse in the past year, use of medications for urinary incontinence or affecting skeletal muscles, change in hormonal replacement therapy in the past 6 months, any leakage of stool or mucus, active urinary or vaginal infection in the past 3 months, or any comorbidities or risk factors interfering with the study.^13^

Participants’ randomization was stratified by center (Montreal and Sherbrooke) and by urinary incontinence type (stress and mixed) within each center. Before the trial, an independent statistician provided a computer-generated list for each of the 4 resulting strata (center by type of urinary incontinence) to create random permutated blocks of varying sizes (4-6). The randomization process took place after the participant’s initial evaluation and written consent. Concealed randomization lists were used by an independent individual to assign eligible participants to 1 of the 2 trial arms (1:1). Research assistants (1 in each center) contacted the independent party to obtain the next sequential randomization and informed participants of their treatment randomization. Study investigators and physiotherapists assessing outcomes were not involved in treatment and remained blinded to the participants’ intervention randomization. Participants were asked not to discuss their intervention with the outcome assessor.

After an individual session with a physiotherapist to learn how to effectively contract the pelvic floor muscle (PFM), women in both treatment arms received a 12-week PFMT program under the direction of an experienced pelvic floor physiotherapist, either in individual or group sessions. For both interventions, each weekly session lasted 1 hour and included a 15-minute educational period and a 45-minute exercise component. The exercise targeted PFM strength, power, endurance, coordination, and integration into daily living activities, such as coughing. The 12-week training protocol comprised three 4-week phases with the gradual addition of increasingly difficult exercises in terms of duration, number of repetitions, and position.^13^ The complete PFMT program is presented in eTable 1 and eTable 2 in Supplement 2.

In addition to the standard protocol, participants in the group-based PFMT arm who reported having difficulty with the PFM exercises were offered short private sessions with the physiotherapist to ensure understanding and correct performance of a PFM contraction. Furthermore, as per standard practice, participants in the individual PFMT arm used intravaginal electromyographic biofeedback during each treatment session for 10 to 15 minutes.

Women in both study arms were expected to perform PFM exercises at home, 5 days per week during the 12-week physiotherapy program, and subsequently 3 days per week for 9 months. Attendance at PFMT sessions was monitored by the treating physiotherapists. Adherence to the home exercise program was assessed through participants’ exercise diaries during the 12-week intervention and then by telephone follow-ups at 6, 9, and 12 months. Participants were asked to refrain from seeking other forms of treatment during the study period (ie, until after the 1-year assessment).^13^

Measurements were taken before the intervention, immediately after the 12-week PFMT period, and 1 year postrandomization. The primary outcome measure was the percentage reduction in the number of urinary incontinence episodes at 1 year reported in a 7-day bladder diary relative to the pretreatment baseline.^18^ Secondary outcomes, assessed in exploratory analyses after the 12-week intervention and at 1 year, included (1) number of daily urinary leakages,^18^ (2) number of micturitions per day and night recorded in the 7-day bladder diary,^18^ (3) amount of leakage on the 24-hour pad test,^19^ (4) 5 International Consultation on Incontinence Questionnaire (ICIQ) modules on urinary incontinence–related symptoms and QoL^20,21^ (ICIQ-Urinary Incontinence Short Form, ICIQ-Nocturia, ICIQ-Vaginal Symptoms, ICIQ-Female Lower Urinary Tract Symptoms Sex, and ICIQ-Lower Urinary Tract Symptoms Quality of Life), (5) Geriatric Self-Efficacy Index,^22^ (6) Patient Global Impression of Improvement questionnaire,^23^ and (7) satisfaction with treatment.^24^ Pelvic floor muscle morphometry and function, PFMT-specific self-efficacy, detailed adherence data, and intervention costs were obtained and will be presented elsewhere.

At 1 year, the proportion of participants reaching a minimal clinically important difference for key urinary incontinence–specific outcomes was compared. In addition, the effectiveness of both interventions was compared in subgroups of interest: center, urinary incontinence type, urinary incontinence severity, age, and BMI. Complications and adverse effects were recorded during the intervention, immediately posttreatment, and at 1 year.

Sample size calculations followed the Consolidated Standards of Reporting Trials (CONSORT) guidelines for randomized noninferiority clinical trials.^25^ The margin of noninferiority was set at a maximum 10% difference between average percentage reductions in urinary incontinence episodes in the individual minus the group-based intervention arms.^26,27^ Accordingly, we calculated the sample size necessary to ensure 90% power for the 95% CI for the intervention effect to exclude a difference higher than 10% when assuming that the true effectiveness of the 2 interventions was equal.^25^ Based on prior evidence, we assumed a within-group SD of the individual PFMT percentage reduction in urinary incontinence episodes scores of 27%.^14,28^ Under these assumptions, accounting for up to 15% attrition rate at 1 year, calculations using the PASS program for noninferiority trials^29^ indicated the need to randomize 182 participants per trial arm.

### Statistical Analysis

Baseline characteristics of the treatment arms were summarized with descriptive statistics. Following CONSORT guidelines for noninferiority trials, main analyses used a per-protocol approach.^25^ In line with the International Consultation on Incontinence recommendations for pelvic floor physiotherapy research,^8^ we focused on outcomes at 1 year.^13^ Therefore, per-protocol analyses were limited to women who completed the 1-year assessment. As recommended, in sensitivity analyses we used the intention-to-treat (ITT) approach^25^ and used the same methods to analyze data on all initially randomized participants, with the last observation carried forward for those who did not complete the 1-year assessments.

Initially, we planned to test the noninferiority hypothesis using the parametric, univariate, independent-sample *t* test to compare the mean outcomes in the 2 trial arms at 1 year and multivariable linear regression models if potential confounders were imbalanced.^13^ However, the observed values of the primary outcome (percent reduction in urinary incontinence episodes) had irregular and skewed distributions and diverged substantially from the classic normal distribution assumed when applying *t* tests and linear regression. Thus, these parametric analyses would be inappropriate for our data, with group-specific means affected by extreme values, resulting in inaccurate 95% CIs and *P* values. Therefore, our primary analyses relied on the nonparametric Wilcoxon rank sum test that compared the median outcome values in the 2 arms and avoided the normality assumption. Accordingly, as recommended for noninferiority trials, the hypothesis of noninferiority was to be accepted if the upper bound of the 2-tailed 95% CI (implying a conservative type I error of 0.025) for the median difference in percentage reduction of urinary incontinence episodes, estimated using a nonparametric bootstrap approach based on 300 resamples, excludes the noninferiority threshold.^25^ In addition, mean values and corresponding *t* test results are reported to assess whether the general pattern of results and final conclusions were consistent between nonparametric and parametric analyses, which addresses statistical significance or nonsignificance of the differences between the 2 trial arms.

We used a multivariable logistic model to compare the odds of achieving a 50% or greater reduction in urinary incontinence episodes at 1 year between group-based and individual PFMT for all participants and for predefined strata based on center, urinary incontinence type, urinary incontinence severity, age, and BMI. Two-way interactions of these stratification variables with the randomization group were tested to verify whether the intervention effect varied across the corresponding strata, with 2-tailed *P* values <.05 considered statistically significant.^30^ Statistical analysis was conducted using R, version 3.1.1 (R Project for Statistical Computing).

## Results

### Trial Participants

**Figure 1.**
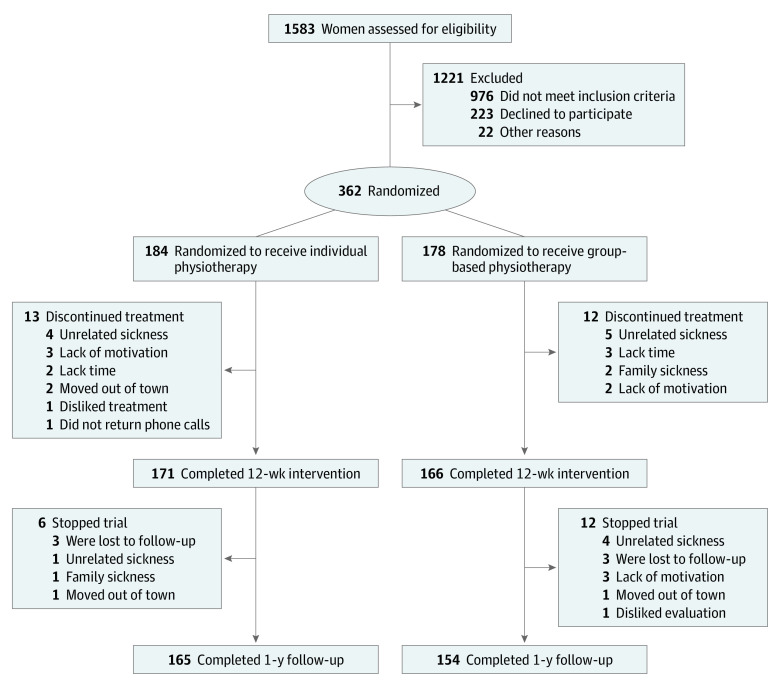
Consolidated Standards of Reporting Trials (CONSORT) Diagram

A total of 362 participants were randomized to either individual PFMT (n = 184) or group-based PFMT (n = 178). Among those, 165 of 184 women (90%) of the individual and 154 of 178 women (87%) of the group-based PFMT completed the 1-year follow-up and were included in the per-protocol analysis (Figure 1). The 43 participants who discontinued the study were included only in the ITT analyses. Those who dropped out had generally similar clinical and demographic characteristics to those who completed the trial, except for the completers being older (mean [SD], 68.2 [5.9] vs 65.8 [4.3] years; *P* = .002) (eTable 3 in Supplement 2).

At baseline, no imbalances in any potential confounders were found between intervention arms (Table 1). The attendance to the 12 PFMT sessions was, on average, 98% (mean [SD], 11.7 [0.6] sessions) for individual and 95% (mean [SD], 11.4 [1.0] sessions) for group-based PFMT. Only 7% (12 of 166) of group-based participants who completed the intervention requested a 20-minute session with their physiotherapists to confirm PFM contraction. Home PFM exercises were performed 4 to 5 times per week by 89% (152 of 171) of the individual and 86% (142 of 166) of the group-based PFMT participants during the 12-week intervention period. Thereafter, PFM exercises were performed at least once per week by 67% (110 of 165) the individual and 69% (107 of 154) of the group-based PFMT participants who completed the 1-year follow-up.

**Table 1.** Baseline Characteristics of the Intention-to-Treat Population

Characteristic	Physiotherapy^a^	
	Individual (n = 184)	Group (n = 178)
Age, mean (SD), y	67.9 (5.9)	68.0 (5.7)
BMI, mean (SD)	27.2 (4.6)	27.0 (4.5)
Parity, median (IQR)	2 (1-2)	2 (1-3)
Cesarean section, median (IQR)	0 (0-0)	0 (0-0)
Vaginal delivery, median (IQR)	2 (0-2)	2 (0.75-3)
Type of incontinence, No. (%)		
Stress	27 (15)	35 (20)
Mixed	157 (85)	143 (80)
Duration of symptoms, mean (SD), y^b^		
Mean (SD)	10.3 (10.6)	9.2 (9)
No. of comorbidities^c^^,^^d^		
Mean (SD)	3.4 (2)	3.5 (2)
No. of medications, mean (SD)	3.2 (2.3)	2.9 (2)
MMSE^e^	29.0 (1.1)	29.1 (1.1)
Previous surgery for incontinence, No. (%)	5 (3)	6 (3)
Current smoker, No. (%)^f^	2 (1)	5 (3)

Abbreviations: BMI, body-mass index (calculated as weight in kilograms divided by height in meters squared); IQR, interquartile range; MMSE, Mini-Mental State Examination.

^a^ None of the between-group comparisons was significant at baseline.

^b^ Data available on 181 participants in the individual cohort and 175 patients in the group cohort.

^c^ Data available on 176 participants in the group cohort.

^d^ Number of comorbidities per participant as reported from a standardized list of 28 diseases and conditions: high blood pressure, osteoporosis, hypercholesterolemia, heart disease, stroke, lung disease/asthma, tuberculosis, depression, loss of vision, vascular diseases, renal failure, glaucoma, transplant, gout, fractured hip, breast cancer, rheumatoid arthritis, other arthritis (ie, osteoarthritis), diabetes, deterioration in hearing, epilepsy, migraines, Parkinson disease, HIV, hepatic impairment, stomach ulcers, thyroid disease, and colitis.

^e^ Scores range from 0 to 30, with lower scores (≤17) indicating severe cognitive impairment and higher scores (≥24) indicating no cognitive impairment.

^f^ Data available on 183 participants in the individual cohort.

### Primary Outcome

**Figure 2.**
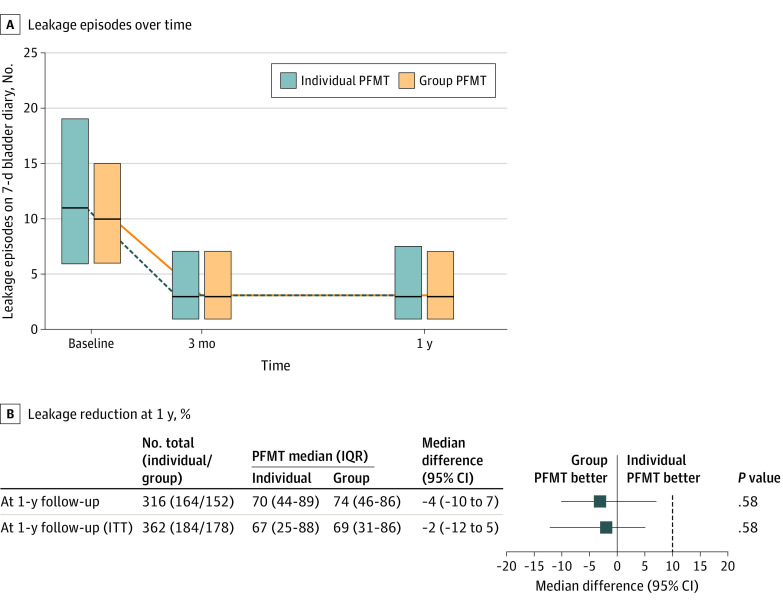
Primary Outcome by Pelvic Floor Muscle Training (PFMT) Treatment Group A, Median frequency and interquartile range (IQR) of leakage episodes at baseline, 3 months, and 12 months following randomization. B, Leakage reduction at 1 year relative to baseline, both per protocol and for the intention to treat (ITT) analysis. In both cases the upper boundary of the 95% CI for the difference in percentage reduction in leakage episodes at 1 year was less than the prespecified margin for noninferiority of 10%.

Figure 2A illustrates the reduction in leakage episode frequency at 12 weeks and 1 year, relative to baseline (*P* < .001 for both intervention arms at both time points). At 1 year, the median percentage reduction in urinary incontinence episodes in the individual intervention was 70% (95% CI, 44%-89%) vs 74% (95% CI, 46%-86%) in the group-based intervention (difference, −4%; 95% CI, −10% to 7%; *P* = .58). In the ITT sensitivity analysis, the median percentage reduction in the number of urinary incontinence episodes in the individual intervention was 67% (95% CI, 25%-88%) vs 69% (95% CI, 31%-86%) in the group-based intervention (difference, −2%; 95% CI, −12% to 5%; *P* = .58). In both of these analyses, the upper boundary of the 95% CI for the difference in percentage reduction in urinary incontinence episodes at 1 year was lower than the prespecified margin for noninferiority of 10% (Figure 2B). Comparison of the mean reductions confirmed a lack of difference with somewhat wider 95% CIs owing to highly nonnormal distributions (per protocol difference, −1%; 95% CI, −13% to 12%; *P* = .54) (eTable 4 in Supplement 2).

### Secondary Outcomes

Table 2 summarizes secondary outcome results related to urinary incontinence. Both of the study arms showed statistically significant improvement for all outcomes relative to baseline. For example, the leakage episodes per day went from a median of 1.6 (interquartile range, 0.9 to 2.7) at baseline to 0.4 (interquartile range, 0.1 to 1.0) at 1 year; *P*<.001 for individual and from 1.4 (interquartile range, 0.9 to 2.1) at baseline to 0.4 (interquartile range, 0.1 to 1.0) at 1 year; *P*<.001 for group-based PFMT). Except for slightly more severe symptoms in the group-based arm immediately after treatment (statistically but not clinically significant difference in median ICIQ-Urinary Incontinence Short Form scores), there were no significant differences between the 2 treatment arms for signs, symptoms, or urinary incontinence–specific QoL outcomes (Table 2). ITT sensitivity analysis confirmed these results (Table 2). Comparisons of the mean values of urinary incontinence–related secondary outcomes showed similar results (eTable 4 in Supplement 2). Moreover, at the 1-year follow-up, a high and almost identical proportion of women in each study arm reported feeling much better or very much better on the Patient Global Impression of Improvement (138 of 163 [85%] vs 132 of 153 [86%]; difference, −1%; 95% CI, −9% to 7%; *P* = .88). Satisfaction with treatment (does not need other treatment) was reported by 148 of 164 (90%) individual vs 139 of 153 (91%) group-based PFMT participants (difference, −1%; 95% CI, −7% to 5%; *P* > .99).

**Table 2.** Urinary Incontinence-Specific Secondary Outcomes at 12-Week and 1-Year Follow-up

Variable	Total participants (individual/group), No.	Median (IQR)		Median difference (95% CI)	*P* value
		Individual PFMT	Group PFMT		
**Leakage episodes/d**					
Baseline	360 (183/177)	1.57 (0.86 to 2.71)	1.43 (0.86 to 2.14)	NA	
After 12-wk treatment	336 (171/165)	0.43 (0.14 to 1.00)	0.43 (0.14 to 1.00)	0 (−0.14 to 0.14)	.20
At 1-y follow-up	318 (165/153)	0.43 (0.10 to 1.00)	0.43 (0.14 to 1.00)	0 (−0.29 to 0.14)	.67
At 1-y follow-up (ITT)	360 (183/177)	0.43 (0.14 to 1.14)	0.43 (0.14 to 1.14)	0 (−0.29 to 0.14)	.57
**Urine loss on pad test, g per 24 h**					
Baseline	351 (180/171)	6.67 (2.42 to 16.05)	5.71 (2.52 to 17.66)	NA	
After 12-wk treatment	319 (166/153)	2.4 (1.16 to 5.82)	2.52 (1.51 to 5.15)	−0.12 (−0.81 to 0.81)	.49
At 1-y follow-up	273 (142/131)	2.75 (1.19 to 6.26)	2.11 (1.10 to 4.86)	0.64 (−0.14 to 1.42)	.92
At 1-y follow-up (ITT)	358 (183/175)	2.97 (1.29 to 7.07)	2.43 (1.24 to 5.59)	0.54 (−0.22 to 1.38)	.85
**ICIQ-UI SF**^**a**^					
Baseline	361 (184/177)	12.50 (10.00 to 14.00)	12.00 (10.00 to 15.00)	NA	
After 12-wk treatment	335 (171/164)	6.00 (4.00 to 8.00)	7.00 (4.75 to 10.00)	−1 (−2 to 0)	.02
At 1-y follow-up	314 (162/152)	7.00 (5.00 to 10.00)	6.00 (4.00 to 10.00)	1 (−1 to 2)	.87
At 1-y follow-up (ITT)	362 (184/178)	7.00 (5.00 to 11.00)	7.00 (4.00 to 11.00)	0 (−0.5 to 2)	.85
**ICIQ-LUTS QoL**^**b**^					
Baseline	358 (182/176)	32.00 (28.00 to 38.00)	32.00 (27.00 to 41.00)	NA	
After 12-wk treatment	336 (171/165)	24.00 (21.00 to 26.50)	24.00 (21.00 to 27.00)	0 (−2 to 1)	.39
At 1-y follow-up	314 (163/151)	23.00 (21.00 to 27.00)	23.00 (21.00 to 27.50)	0 (−1 to 1)	.51
At 1-y follow-up (ITT)	361 (184/177)	24.00 (21.00 to 27.00)	23.00 (21.00 to 29.00)	1 (−1 to 1.5)	.52
**Perceived benefit on PGI-I, No. (%)**^**c**^					
After 12-wk treatment	337 (171/166)	164 (96)	160 (96)	0 (−4 to 4)	>.99
At 1-y follow-up	316 (163/153)	138 (85)	132 (86)	−1 (−9 to 7)	.88
At 1-y follow-up (ITT)	337 (171/166)	146 (85)	144 (87)	−2 (−9 to 5)	.91
**Satisfaction, No. (%)**^**d**^					
After 12-wk treatment	336 (171/165)	160 (94)	150 (91)	3 (−3 to 9)	.48
At 1-y follow-up	317 (164/153)	148 (90)	139 (91)	−1 (−7 to 5)	>.99
At 1-y follow-up (ITT)	336 (171/165)	154 (90)	150 (91)	−1 (−7 to 5)	.94

Abbreviations: ICIQ-UI SF, International Consultation on Incontinence Modular Questionnaire-Urinary Incontinence Short Form; ICIQ-LUTS QoL, International Consultation on Incontinence Modular Questionnaire-Lower Urinary Tract Symptoms Quality of Life; IQR, interquartile range; ITT, intention-to-treat; NA, not applicable; PFMT, pelvic floor muscle training; PGI-I, Patient Global Impression of Improvement.

^a^ Score range, 0 to 21; greater values indicate increased severity (minimal clinically important difference, 2.52 points).^27^

^b^ Score range, 19 to 76; greater values indicate a higher effect on quality of life (minimal clinically important difference, 3.71 points).^27^

^c^ Number of participants stating they are very much better or much better.

^d^ Number of participants stating they were satisfied (do not need another treatment) as opposed to unsatisfied (would like another treatment).

For all secondary outcomes related to other lower urinary tract symptoms, there were no statistically significant differences between the 2 treatment arms at the 1-year follow-up in both per-protocol and ITT analysis in either the median (Table 3) or the mean values (eTable 5 in Supplement 2). Urinary frequency and nocturia (>2 episodes per night) on the 7-day bladder diary and the ICIQ-Nocturia questionnaire showed statistically significant improvements relative to baseline at both time points in each of the 2 arms. Urinary frequency per day went from a median of 8.3 (IQR, 6.3-10.1) micturitions at baseline to 7.4 (6.1-8.7) (*P* < .005) at 1 year for individual and from 8.5 (IQR, 6.9-10.1) at baseline to 7.0 (IQR, 5.9-8.4) (*P* < .005) at 1 year for group-based PFMT. The number of participants with nocturia (>2 episodes per night) went from 59 participants (32%) at baseline to 36 participants (22%) (*P* = .04) at 1 year for individual and from 68 (38%) at baseline to 38 (25%) (*P* < .005) at 1 year for group-based PFMT. All 3 ICIQ-Vaginal Symptoms subscales (vaginal symptoms, sexual matter, and vaginal symptoms effect on QoL) were significantly improved at both time points. For example, vaginal symptoms subscale on the ICIQ-VS went from a median of 4.0 (IQR, 0.0- 9.5) at baseline to 2.0 (IQR, 0.0-5.5) (*P* < .005) at 1 year for individual and from 4.0 (IQR, 1.0-10.0) at baseline to 0.5 (IQR, 0.0-4.3) (*P* < .005) at 1 year for group-based PFMT. The participants’ confidence in their ability to prevent urine loss according to the Geriatric Self-Efficacy Index also improved: the total score went from a median of 55 (IQR, 41-69) at baseline to 89 (IQR, 70-102) (*P* < .005) at 1 year for individual PFMT and from 59 (IQR, 44-73) at baseline to 93 (IQR, 76- 107) (*P* < .005) at 1 year for group-based PFMT. However, findings on sexual issues associated with lower urinary tract symptoms as determined by the ICIQ-Female Lower Urinary Tract Symptoms Sex did not change significantly at any time.

**Table 3.** Further Secondary Outcomes at 12-Week and 1-Year Follow-up

Variable	Total participants (individual/group), No.	Median (IQR)		Median difference (95% CI)	*P* value
		Individual PFMT	Group PFMT		
**Other lower urinary tract symptoms**						
Micturition, 7-d bladder diary					
Baseline	359 (183/176)	8.29 (6.29 to 10.14)	8.5 (6.86 to 10.14)	NA	
After 12-wk treatment	336 (171/165)	6.86 (5.57 to 8.29)	6.57 (5.57 to 8.14)	0.29 (−0.43 to 0.71)	.63
At 1-y follow-up	318 (165/153)	7.43 (6.14 to 8.71)	7.00 (5.86 to 8.43)	0.43 (−0.14 to 0.86)	.88
At 1-y follow-up (ITT)	360 (183/177)	7.29 (6.07 to 8.64)	7.00 (5.71 to 8.43)	0.29 (−0.14 to 0.86)	.86
Nocturia, >2 episodes per night, No. (%)					
Baseline	362 (184/178)	59 (32)	68 (38)	NA	
After treatment	337 (171/166)	29 (17)	35 (21)	−4.00 (−12.00 to 4.00)	.57
1-y follow-up	317 (164/153)	36 (22)	38 (25)	−3.00 (−12.00 to 6.00)	.24
At 1-y follow-up (ITT)	361 (184/177)	38 (21)	46 (26)	−5.00 (−14.00 to 4.00)	.12
ICIQ-N^a^					
Baseline	361 (184/177)	2.00 (1.00 to 4.00)	3.00 (2.00 to 4.00)	NA	
After treatment	336 (170/166)	1.00 (1.00 to 2.00)	1.00 (1.00 to 2.00)	0.00 (−1.00 to 1.00)	.13
1-y follow-up	317 (164/153)	2.00 (1.00 to 3.00)	2.00 (1.00 to 2.00)	0.00 (−1.00 to 1.00)	.40
At 1-y follow-up (ITT)	362 (184/178)	2.00 (1.00 to 3.00)	2.00 (1.00 to 3.00)	0.00 (−1.00 to 0.50)	.25
**Vaginal and sexual symptoms**						
ICIQ-VS vaginal symptoms subscale^b^					
Baseline	358 (183/175)	4.00 (0.00 to 9.50)	4.00 (1.00 to 10.00)	NA	
After 12-wk treatment	335 (170/165)	2.00 (0.00 to 6.00)	2.00 (0.00 to 6.00)	0.00 (−1.00 to 2.00)	.77
At 1-y follow-up	315 (163/152)	2.00 (0.00 to 5.50)	0.50 (0.00 to 4.25)	1.50 (0.00-3.00)	.89
At 1-y follow-up (ITT)	362 (184/178)	2.00 (0.00 to 6.00)	2.00 (0.00 to 6.00)	0.00 (−1.50 to 2.00)	.67
ICIQ-VS sexual matters subscale^c^					
Baseline	127 (70/57)	0.00 (0.00 to 25.50)	0.00 (0.00 to 36.00)	NA	
After 12-wk treatment	120 (63/57)	0.00 (0.00 to 5.50)	0.00 (0.00 to 28.00)	0.00 (−9.00 to 0.00)	.05
At 1-y follow-up	103 (58/45)	0.00 (0.00 to 10.25)	0.00 (0.00 to 8.00)	0.00 (0.00-4.00)	.81
At 1-y follow-up (ITT)	160 (87/73)	0.00 (0.00 to 13.50)	0.00 (0.00 to 24.00)	0.00 (−3.00 to 1.00)	.39
ICIQ-VS quality of life subscale^d^					
Baseline	361 (184/177)	0.00 (0.00 to 3.00)	0.00 (0.00 to 4.00)	NA	
After 12-wk treatment	334 (169/165)	0.00 (0.00 to 0.00)	0.00 (0.00 to 0.00)	0.00 (0.00 to 0.00)	.46
At 1-y follow-up	314 (163/151)	0.00 (0.00 to 0.00)	0.00 (0.00 to 0.00)	0.00 (0.00 to 0.00)	.80
At 1-y follow-up (ITT)	362 (184/178)	0.00 (0.00 to 0.00)	0.00 (0.00 to 0.00)	0.00 (0.00 to 0.00)	.47
ICIQ-FLUTS^e^					
Baseline	347 (176/171)	8.00 (2.00 to 8.00)	8.00 (3.00 to 8.00)		
After 12-wk treatment	325 (161/164)	8.00 (1.00 to 8.00)	8.00 (1.00 to 8.00)	0.00 (−3.00 to 0.00)	.46
At 1-y follow-up	309 (161/148)	8.00 (1.00 to 8.00)	8.00 (2.00 to 8.00)	0.00 (0.00 to 0.00)	.14
At 1-y follow-up (ITT)	360 (184/176)	8.00 (1.00 to 8.00)	8.00 (2.00 to 8.00)	0.00 (0.00 to 0.00)	.20
**Self-efficacy**						
Geriatric self-efficacy index^f^					
Baseline	362 (184/178)	55.00 (41.00 to 69.00)	59.00 (44.25 to 73.00)	NA	
After 12-wk treatment	335 (170/165)	92.00 (79.00 to 104.75)	95.00 (80.00 to 107.00)	−3.00 (−8.00 to 3.00)	.80
At 1-y follow-up	309 (159/150)	89.00 (70.00 to 102.00)	93.00 (76.25 to 107.00)	−4.00 (−12.50 to 2.00)	.98
At 1-y follow-up (ITT)	362 (184/178)	86.50 (69.00 to 102.00)	92.00 (74.00 to 106.75)	−5.50 (−12.50 to 1.50)	.95

Abbreviations: ICIQ, International Consultation on Incontinence Questionnaire; ICIQ-FLUTS, ICIQ-Female Lower Urinary Tract Symptoms Associated With Sexual Matters; ICIQ-N, ICIQ-Nocturia; ICIQ-VS, ICIQ-Vaginal Symptoms; IQR, interquartile range; ITT, intention to treat; LUT, lower urinary tract; PFMT, pelvic floor muscle training.

^a^ Score range, 0 to 8; higher values indicate increased symptom severity.

^b^ Score range, 0 to 53; higher values indicate increased symptom severity.

^c^ Score range, 0 to 58; higher values indicate increased symptom severity.

^d^ Score range, 0 to 10; higher values indicate increased symptom severity.

^e^ Score range, 0 to 14; higher values indicate increased symptom severity.

^f^ Score range, 0 to 120; higher values indicate higher self-efficacy.

A high and almost identical proportion of women reached the minimal clinically important difference for leakage episodes, urinary incontinence severity, and urinary incontinence–specific QoL at the 1-year follow-up in the 2 arms (eTable 8 in Supplement 2). In addition, multivariable logistic analyses of the 1-year primary dichotomized outcome (percentage of urinary incontinence reduction <50% or ≥50%) showed that, after Bonferroni correction owing to multiple interactions to test, there were no statistically significant interactions between the intervention and any of the a priori identified potential effect modifiers (study center, urinary incontinence type, urinary incontinence severity, age, and BMI) (eTable 6 and eTable 7 in Supplement 2). These results confirmed that the association between the intervention arms (group-based and individual PFMT) and the binary outcome was similar across different subgroups.

### Adverse Events

Throughout the trial, no serious adverse events were reported in either study arm. Minor adverse events were reported by 27 women in the individual PFMT arm, including vaginal spotting (6 women) or vaginal discomfort while using intravaginal biofeedback (21 women). Five women in the group-based PFMT arm reported vaginal discomfort. These reported adverse events occurred primarily in the first 2 sessions and resolved without the need for treatment.

Although the participants were asked to refrain from seeking other forms of treatment specific to their urinary incontinence condition during the study period, 5 of 165 women (3%) in the individual PFMT arm vs 7 of 154 women (5%) in the group-based PFMT arm reported having visited health professionals, taken medication, or pursued other treatments at the 1-year follow-up. Details are given in eTable 9 in Supplement 2.

## Discussion

To our knowledge, this is the first and only adequately powered trial to assess noninferiority of group-based PFMT compared with individual PFMT for urinary incontinence. In both per-protocol and ITT analyses, group-based PFMT was not inferior to the standard individual PFMT for the treatment of stress or mixed urinary incontinence in older women. These results provide controlled evidence that confirms earlier inconclusive findings from 6 smaller randomized clinical trials in younger women. Many of these trials had significant risk of bias, no long-term follow-up, and were not sufficiently powered or designed to assess noninferiority.^31,32,33,34,35,36,37^

Our study demonstrated a median percentage reduction in urinary incontinence episodes at 1 year of 70% in individual PFMT compared with a 74% reduction in group-based PFMT, which did not vary with study center, urinary incontinence type, urinary incontinence severity, age, and BMI. Furthermore, these findings indicate equal or greater urinary incontinence reduction compared with previous studies assessing the effect of individual physiotherapy in a similar population.^8,24,38^ Individual and group-based PFMT had similar effectiveness for all secondary outcomes except sexual issues associated with lower urinary tract symptoms at 1 year. Adherence to treatment sessions and home exercises was high and loss to follow-up was low, indicating acceptability of both interventions. Few participants had adverse events; all were minor, reversible, and occurred primarily in the early stage of each intervention.

### Limitations and Strengths

This study’s exclusion criteria and intensive intervention could limit generalizability of the results to either frail older or younger women who may not be able to participate in or adhere to this intensive intervention. From a clinical and health service perspective, strengths of our findings indicate that the group-based approach makes it possible to rapidly increase the number of women treated with PFMT. Making conservative management more accessible may help delay or reduce the need for urinary incontinence surgery and reduce the burden of urinary incontinence on the health care system. Overall, our results demonstrate that older women with stress or mixed urinary incontinence will attend, adhere, and gain clinically important benefits from group-based PFMT.

## Conclusions

In a multicenter noninferiority trial, group-based PFMT was shown to be noninferior to the standard individual PFMT at 1 year postrandomization for the treatment of stress and mixed urinary incontinence in older women. Widespread use of this effective intervention could positively affect continence-care affordability and treatment availability.

## Notes

Supplement 1. Trial Protocol

Supplement 2. eTable 1. Consensus of Exercise Reporting Template (CERT) eTable 2. Supervised Pelvic Floor Muscle Training Program eTable 3. Baseline Characteristics of the Population That Completed the Study vs the Population That Did Not Complete the Study eTable 4. Mean Values for the Primary and Urinary Incontinence Specific Secondary Outcomes in Individual and Group-Based Pelvic Floor Muscle Training at 12-Weeks and One-Year Follow-up eTable 5. Mean Values for the Impact On Other Lower Urinary Tract Symptoms, Vaginal, Sexual Symptoms and Self-Efficacy in Individual and Group-Based Pelvic Floor Muscle Training at 12-Weeks and One-Year Follow-up eTable 6. Primary Outcome- Percentage Reduction of Leakage Episodes in Individual and Group-Based Interventions at One-Year Follow-up According to Subgroups of Interest eTable 7. Odds Ratios for the Association Between the Intervention Arm (Group-Based Pelvic Floor Muscle Training vs Individual Pelvic Floor Muscle Training) and the Binary Outcome of Percentage Reduction of Leakage Episodes eTable 8. Minimal Clinically Important Differences at the One-Year Follow-up eTable 9. Other Treatments Taken During the Study Period eReferences

Supplement 3. Data Sharing Statement
