# Supplementary material for: Group-Based vs Individual Pelvic Floor Muscle Training to Treat Urinary Incontinence in Older Women: A Randomized Clinical Trial
Source: JAMA Intern Med. 2020 Aug 3;180(10):1284–93. doi: 10.1001/jamainternmed.2020.2993 (PMC7400216; doi:10.1001/jamainternmed.2020.2993)
Supplement: Supplement 1. — Trial Protocol [file jamainternmed-e202993-s001.pdf]

Group-based versus individual pelvic floor muscle training to treat urinary incontinence in older women: the Group trial protocol

The recommended treatment for urinary incontinence (UI) in women is individual pelvic floor muscle training (PFMT). Canada is currently unable to meet the demand for this costly and resource-intense approach. This proposal seeks funding for a five-year randomized controlled trial to determine if the effectiveness of group-based physiotherapy is not inferior to individual pelvic floor physiotherapy in women age 60 and over with stress or mixed UI, and to establish the cost-effectiveness of both. Demonstrating that group-based treatment is at least as effective as individual one-on-one treatment and more cost-effective would justify recommending group-based PFMT as first-line treatment for UI.

1. CONTEXT

UI is *"any involuntary leakage of urine"*.[1] UI is one of the most prevalent health concerns confronting women age 60 and over.[2,3] Up to 55% of community-dwelling women in this age group suffer from UI, with 20 to 25% of these women experiencing severe symptoms (>10 episodes/week).[2-4] There are three symptom types: urge (*involuntary leakage accompanied by or immediately preceded by an urgent need to urinate*); stress (*involuntary leakage related to effort, exertion, sneezing or coughing*); and mixed (*urge and stress UI symptoms*) UI.[1] The ratio of UI types differs according to age group, with older women predominantly affected by mixed and stress UI.[3]

UI affects a large number of women, and the prevalence is increasing. In Canada, the percentage of women age 60 and over is expected to grow rapidly over the next 15 years, from 16% in 2011 to an estimated 23% by 2026.[5] A corresponding increase in UI prevalence and severity is anticipated, which in turn will increase the number of women requiring incontinence treatment. Moreover, the leading edge of the baby boomer population has now reached age 60, and the demand for UI treatment is expected to continue to grow.

UI is a serious medical condition that impinges on other medical conditions. UI can lead to urinary tract infections, pressure ulcers and perineal dermatosis.[6,7] It engenders significant social problems, embarrassment and negative self-perception,[8,9] and reduces social interactions and physical activities.[8,9] It is associated with poor self-rated health,[9] impaired emotional and psychological well-being,[8,10] impaired sexual relationships, and increased nursing home admissions.[6,8-12] Women with UI, particularly older women, often find themselves isolated and relatively inactive in the medium or long term.[10] **Given the importance of physical activity for the prevention of costly vascular diseases,** cancer, osteoporosis and dementia, any condition that negatively impacts physical activity must be addressed.[10,13-16]

UI costs are significant and will increase. UI is not only costly to individuals but also to the Canadian healthcare system. Incontinence costs Canadians approximately $5.6 billion per year.[17] Individuals with incontinence spend on average $1,500 per year on protection products alone. Incontinence also costs the Canadian healthcare system $1.9 billion per year.[17] With over two million older women at risk of UI within the next 15 years, healthcare professionals and administrators alike will be called to provide access to more cost-effective treatments.

UI can be treated effectively. There are three categories of UI treatment: conservative, pharmacological and surgical – the latter two being the most invasive and expensive, and hence not recommended as first-line treatment.[18-20] Conservative treatments include PFMT, lifestyle intervention and bladder retraining.[20] National and international clinical practice guidelines such as those from the Canadian Obstetrics and Gynecology Association,[21] the Canadian Urological Association[22] and the Fourth International Consultation on Incontinence[20] recommend supervised PFMT as first-line treatment for stress and mixed UI in women (level of evidence: A). With the supervision of a trained healthcare professional, progressive individual PFMT, with or without adjunctive biofeedback, is used to improve pelvic floor muscle (PFM) function.[20] Systematic reviews and more than 88 randomized controlled trials (RCTs) support the effectiveness of supervised PFMT for UI in young, middle-aged and older women.[23,24] Among these studies, five trials exclusively targeted older community-dwelling women,[25-29] who were experiencing stress [25-27] or mixed UI.[28,29] Both types of UI responded to individual PFMT, resulting in higher continence rates, with 56% to 70% of participants reporting continence post treatment.[25-29] **These studies clearly demonstrate that older women with both types of UI respond to the first-line treatment, individual PFMT. However, high costs and insufficient human resources impede access.**

Despite the negative impacts of UI and the evidence supporting the efficacy of PFMT, less than a third of older women with UI receive treatment.[30-32] According to studies on treatment barriers, major barriers include the lack of adequate human and material resources to address this problem through one-on-one physiotherapy sessions and the inability

to identify which women are most likely to benefit from PFMT in pre-treatment.[17,33-35] For example, only 264 of Quebec's 4230 physiotherapists, or 6.2%, are qualified to conduct PFMT. Of these, only 72 (27.3%) practice in hospitals where access is limited by waiting lists of six months to one year. The remaining 192 practitioners offer treatment through private practice at rates of $70 to $90 per treatment. However, with six-month waiting lists, these practitioners are unable to accommodate the growing number of referrals (Ordre professionnel de la physiothérapie du Québec, unpublished statistics). Moreover, there is no validated prediction tool capable of linking a patient's individual characteristics and symptoms to the ideal treatment options. Hence, women are uncertain in pretreatment if they will be among those most likely to benefit from PFMT. Furthermore, many women, notably pensioners, cannot afford the cost of a 12-week program, which is typically in the range of $840-$1080. These factors cause more women to opt for more costly surgical options, which are covered by the Canadian healthcare system.[17] **In its briefing document for policy-makers, the Canadian Continence Foundation urges the government to increase funding for conservative treatments in light of forward-thinking fiscal and economic management.**[17]

Evidence from two recent RCTs (2011) suggests that group PFMT is effective for treating stress and mixed UI in older women, resulting in high continence rates (60%) post-intervention, as compared to no treatment[36] or bladder training.[37] This new group-delivery approach offers an effective way to overcome financial and human resource barriers. Moreover, it has been shown to increase participant motivation, compliance and UI self-management capabilities.[38-42] In recent years, the search for less costly forms of rehabilitation treatments has taken on a greater impetus and been subject to intense debate.[43] It is now recognized that for some pathological conditions, group rehabilitation approaches offer a viable solution, one that would permit better allocation of available economic, material and human resources.[43] Additionally, group intervention is already acknowledged within the field of health promotion as a powerful tool supporting behavior modification. Group sessions provide greater motivation (by reducing an individual's sense of isolation) as well as a forum for providing information (particularly for patients who are hesitant to ask questions), and foster peer support and discussions (e.g., Weight Watchers, Alcoholics Anonymous).[44] Tackling UI through group sessions could also prove to be an effective means of educating and encouraging active self-management.[38-41,43,44]

Currently, standard individual PFMT has been shown to be effective in the short term (less than six months), with gradual symptom reoccurrence over the long term.[45] Research indicates that adherence to PFMT exercises at home post-treatment improves mid- to long-term effectiveness.[46] Self-efficacy, motivation and support are also key factors influencing adherence.[46]

Hence, group PFMT has the potential to address not only resource issues but also sustain the effectiveness of treatment in the medium and long term, further increasing its cost effectiveness as a UI treatment. Thus, group PFMT could prove to be an excellent, cost-effective treatment for Canada's growing population of older UI-afflicted women.

Study led by an experienced UI research team The Principal Investigator (PI) has undertaken extensive research in UI, including oversight of a 2002-2009 RCT, ***Physiotherapy for persistent post-natal stress urinary incontinence: a randomized controlled trial*** (CIHR grant No.36053), comparing the effectiveness of individual PFMT to no treatment in women with persistent postnatal stress UI. The RCT showed individual PFMT to be effective in reducing UI in 78% of treated women.[47] A seven-year follow-up study demonstrated that 50% of the women were still continent. Notably, those adhering to PFMT over the seven years did significantly better.[45-48] **From 2007-2009,** this research team conducted a pilot project assessing the feasibility of group PFMT as a treatment for UI in women age 60 and over: ***Pelvic floor exercise classes for urinary incontinence in older women: a feasibility study*** (Réseau québécois de recherche sur le vieillissement-FRSQ grant).[42,49] The study assessed the feasibility of employing group PFMT to treat older women (age 60+) with stress and mixed UI. Twenty-seven participants were split into groups of six to eight people, and attended 12 group-based PFMT classes over the course of two months. The assessments and interventions were well tolerated, and the attrition rate was below 10%. Participants complied with study demands in terms of attendance at treatment sessions (90%), completion of the daily exercise program (78%) and data collection (95%). Post-study focus groups identified "*close supervision by the physiotherapist*" and *"short daily PFM exercises"* as key facilitators in sustaining the subjects' weekly participation in the exercise classes, completion of the 12-week program and adherence to the daily home exercise program. Incontinence episodes were reduced by 50% per week.[42,49] This study also demonstrates the feasibility and potential effectiveness of group PFMT to treat UI in older women. **From 2009-2011**, this research team completed a large cohort study aimed at identifying PFM dysfunction in older women with UI (FRSQ: 14417 and CIHR MOP 84529). Preliminary results[50] indicate that maximal contraction strength, coordination and tone were deficient in older women with UI. Previous work enabled the development of a more targeted PFMT approach, which needs to be validated. **Finally, in 2011,** a citizens' jury study was completed involving older women with UI. **Preliminary results identified PFM exercises, education and peer support as the three most highly ranked priority needs. Group-based PFMT addresses all these needs.**

Considering (1) the high prevalence of UI among older women and projected population growth combined with, (2) the

barriers to the use of the recommended first-line treatment, (3) the gradual symptom reoccurrence in long-term post-PFMT, (4) the recently demonstrated effectiveness of group-based PFMT in the short term and the potential added value for long-term effects, and (5) the promising results of the feasibility study, **there is a clear need to compare the impact of group-based to individual PFMT in the treatment of UI in women age 60 and over. It is unlikely that physicians, physiotherapists and women will switch to group-based PFMT without the proposed study.**

2. OBJECTIVES

The overall objective of the GROUP (Group Rehabilitation Or IndividUal Physiotherapy for Urinary Incontinence in Older Women) trial is to determine if group-based PFMT for women age 60 and over with stress or mixed UI is not meaningfully less effective, sustainable and affordable than the currently recommended individual (one-on-one) PFMT. The specific objectives are to compare the effectiveness of group-based PFMT and individual PFMT on:

1. The percent reduction in the number of UI episodes, as measured by the seven-day diary [28], one-year post randomization (primary outcome).

2. Lower urinary tract symptoms, level of distress, and quality-of-life impact, immediately post intervention and one-year post randomization. These secondary outcomes will be measured by, respectively, the seven-day bladder diary (number of UI episodes, number of micturitions),[51] 24-hour pad test (quantity of urine loss)[52] and five modules of the International Consultation on Incontinence Questionnaire (ICIQ): the ICIQ-Urinary Incontinence short form (ICIQ-UI short form),[53] the ICIQ-Nocturia Modile (ICIQ-N),[54] the ICIQ-Lower Urinary Tract Symptoms Quality of Life Module (ICIQ-LUTSquol),[55] the ICIQ-Vaginal Symptoms Module (ICIQ-VS),[56] the ICIQ-Gender-Specific Sexual Matters Module (ICIQ-FLUTSex)[57].

3. Self-efficacy, immediately post intervention and at one-year post randomization, as measured by the Geriatric Self Efficacy Scale[58] and the Broom Self Efficacy Questionnaire (Part A)[59].

4. Impression of improvement and benefits, immediately post intervention and at one-year post randomization, as measured by the Patient Global Impression of Improvement Index (PGI-I)[60] and the Benefit (satisfaction) and willingness to have another treatment (B&W questionnaire)[37];

5. PFM strength, function, and morphometry as measured by the digital Oxford Scale,[61] pelvic floor dynamometry,[62] transperineal ultrasound,[63] immediately post intervention and one-year post randomization.
The direct costs of group-based PFMT and individual PFMT will also be compared using the adapted Dowell–Bryant Incontinence Cost Index (DBICI)[64] immediately post intervention and one-year post randomization.

3. METHODOLOGY

3.1 Trial design

A non-inferiority RCT is proposed for this study conducted in two Quebec centers (Montreal and Sherbrooke). A non-inferiority design was chosen because: 1) individual PFMT is the standard of care, 2) recent literature and preliminary data on older women indicates that PFMT is effective immediately post-treatment and in the short term (less than six months), 3) there are potential long-term benefits from group-based interventions resulting from increased peer-support, mutual self-help and increased compliance with treatment, 4) the anticipated cost is lower, and 5) potential accessibility of care can be improved through a group approach. A change in clinical practice would be justified should this study find that group-based PFMT is at least as effective as individual PFMT.

Population: The target population is older women with UI. "Older" is defined as age 60 and over. This population of post-menopausal women have a distinct UI profile from that of pre-menopausal or postpartum women.[65] Other RCTs on UI in older women also employ this cut-off age.[37,66] There is no upper age limit, as women as old as 98 have been cured of UI through PFMT.[67] Participants will be classified as incontinent if they report a weekly average of three or more episodes of involuntary urine loss during the preceding three months. This is a validated indicator of UI that has been used in cohort studies and RCTs on UI.[36,66] Finally, the type of UI will be confirmed as a pattern of stress/mixed UI on the validated Questionnaire for Incontinence Diagnosis (QUID).[68]

3.1.1 Inclusion and exclusion

Determination of eligibility will be achieved in two steps: 1) telephone eligibility evaluation with a research assistant, and 2) on-site eligibility evaluation with the evaluator.

Women will be included if they 1) are age 60 or older, 2) describe a pattern of stress/mixed UI on the QUID,[69] 3) have at least three urinary leakages per week, persisting for three months or more, 4) understand French or English instruction, and 5) are able to provide informed consent, complete a seven-day bladder diary and have a gynecological examination[70]

Women will be excluded if they present risk factors known to interfere with the effects of PFMT: chronic constipation (as defined by the International Working Committee for Chronic Constipation);[71] obesity (body mass index >35)[72]; experience important organ prolapse (>2 degree Pop-Q)[73] likely to interfere with the PFM evaluation.; have reduced mobility (not mobile without the aid of a cane, crutches or a walker); have any other medical problems or major functional impairments likely to interfere with treatment or evaluation, including active cancer, dementia (Mini-Mental State Exam (MMSE) < 24),[74] diabetes, severe arthritis, serious cardiovascular disease, psychiatric conditions, severe neurological conditions, myopathies or a current illness requiring hospitalization; currently taking any medication for urinary incontinence (eligible if comfortable stopping the medication) or medications affecting skeletal muscles; experiencing any leakage of stool or mucus; have an active urinary or vaginal infection in last three months; are on hormone replacement and their prescriptions have not been stable for at least six months (this criterion is included to eliminate possible changes in urinary status secondary to changes in hormonal status);[75] have had recent UI or organ prolapse surgery (within the last year); and are receiving or have received a physiotherapy intervention for UI within the last year. Patients excluded from the study will be referred to their healthcare provider to receive standard incontinence care.

3.1.2 Recruitment Women will be recruited from the participant bank of the Centre de recherche de l'Institut universitaire de gériatrie de Montréal (CRIUGM), community advertisements, and continence, urology and urogynecological clinics within the metropolis of the two study centers. A poster explaining the research project will be displayed at each clinic. Interested candidates will be invited to call the research coordinator.

a) Telephone eligibility evaluation (20 min.) Initial contact will be made over the phone. A research assistant will briefly explain the project. Potential candidates will be informed of the study's objectives and procedures. If interested, they will be screened for eligibility using a standardized telephone questionnaire developed and used in this research team's previous studies. UI type will be determined by the *Questionnaire for Incontinence Diagnosis* (QUID) over the telephone. QUID is a six-item self-administered questionnaire developed to identify and differentiate UI types in women.[68] This method has shown good internal consistency and test-retest reliability, good content and criterion validity, and good specificity/sensitivity in women with UI.[76] Furthermore, QUID is highly recommended for UI type classification by the International Consultation on Incontinence (Grade A).[76] In older women, both severity of incontinence and UI type have been identified as the most significant factors correlating with treatment response.[76] The consent form will be mailed to participants with the seven-day bladder diary[70,76] and the 24-hour pad test. The diary and pad test will be completed and returned at the onsite eligibility evaluation.

b) Eligibility evaluation (1 hour) Women will be scheduled for onsite evaluations with a physiotherapist if they report at least three UI episodes in the seven-day bladder diary. After recovering the bladder diary and the pad test, and signing the consent form, the MMSE[74] questionnaire will be completed (excludes women with cognitive impairment). A vaginal exam will also be conducted to identify any perineal pain or prolapse likely to interfere with either the evaluation or the intervention**. Participants who are not eligible for the present study will be referred back to their healthcare provider.** Eligible participants will complete the pre-intervention evaluation (section 3.5).

Randomization and blinding To prevent imbalance on important patient characteristics, while ensuring equal sizes of the two trial arms, stratified randomization will be used with random blocks within each stratum. Specifically, participant allocation will be stratified by: (1) center (Montreal and Sherbrooke); and subsequently (2) within each center, by UI type (MUI and SUI). Within each of the four resulting strata (center-by-UI type), the randomization sequence will be generated by a computerized system operated by a CRIUGM statistician before the trial to create random permutated blocks of varying sizes (4–6), making the particular sequences difficult to predict. Randomization will take place after a participant's initial evaluation and written consent. Randomization lists will then used by an independent individual to assign eligible participants to one of the two trial arms. A research assistant (one in each center) will communicate with this person to obtain the next sequential allocation and inform the participants of their treatment allocation. In addition, the research assistant will organize the logistics of the PFMT intervention.

Investigators, data analysts and physiotherapists in charge of outcome evaluations will remain blinded to the individual participant's trial group allocation. Although participants cannot be blinded to their own group allocation, they will be blinded to the study's hypothesis and to the treatment offered in the alternate group. Despite their different formats, both groups will have parallel, weekly sessions in terms of content and time but on different days. To minimize the likelihood of assessor unblinding, participants will be asked not to discuss their treatment with the independent assessor. In addition, the assessments will be separated from the intervention session in timing and location.

Interventions Women in both groups will receive the same 12-week PFMT, either in one-on-one sessions or as part of a group, under the direction of a physiotherapist trained in pelvic floor rehabilitation. The choice of a 12-week PFMT approach is based on muscle physiology theory: strength training programs show positive effects after 8-12 weeks.[77] In post-menopausal women, Gunnarsson reported a significant improvement in strength and reduction in UI episodes after 12 weeks of training.[78] Although there is no known side effect/complication related to PFMT other than possible discomfort following the intervention, any adverse event will be monitored. All participants will be instructed to contact research personnel should they experience any adverse event at any point during the study.

3.2 Physiotherapy sessions, both groups For both groups, the weekly sessions will last one hour and include a 15-minute educational period and a 45-minute exercise component. The educational period will cover the various functions of the PFM, including pre-contraction and normal bladder control, and voiding parameters, such as fluids and fluid intake, toilet positions and voiding dynamics. The exercise component will include strength, endurance and coordination exercises. Between PFM exercises, lower extremity strength, balance and functional exercises (dance) will be performed. This approach is in line with research findings indicating that older women with mixed and stress UI have reduced PFM and lower-extremity strength, and balance compared to continent women.[79] This approach is used for PFMT programs designed for older adults.[36,37]The treatment protocol will be divided into three phases, allowing for the gradual progression of treatment, where increasingly difficult exercises are added in terms of exercise duration, repetition and position. Each phase will last four weeks.

Individual PFMT group: Women in this group will participate in weekly, one-on-one, one-hour sessions for 12 consecutive weeks with an experienced physiotherapist. Each session will consist of a 15-minute educational period followed by a 45-minute PFMT program as described above. Participants in the group-based PFMT will also receive one to three private sessions with the physiotherapist leading their group to ensure that each participant understands how to correctly perform a PFM contraction, as confirmed by vaginal digital palpation.[37]

Group-based PFMT group Women in this group will participate in groups of eight in one-hour weekly PFMT sessions under the supervision of an experienced physiotherapist. Each session will consist of a 15-minute educational period followed by a 45-minute PFMT program as described above; however, the PFMT exercises will be conducted within a group. The PFM exercise class developed by Bo[80] has been adapted, based on this research team's 2009 pilot project,[42,49] in order to render it more feasible for older women. This protocol is also consistent with this research team's more recent cohort study (FRSQ 14417), indicating it is both feasible and effective.[81] **Both individual and group-based sessions will be conducted at CRIUGM in Dr Dumoulin's laboratory or at the Centre de recherche clinique Étienne-Le Bel (CRCEL) du Centre hospitalier universitaire de Sherbrooke in Dr Morin's laboratory.**

Home PFM exercise program Women in both groups will be expected to perform PFM strength, endurance and coordination exercises at home, five days per week, for the duration of the treatment.[42,49] To support treatment progression, the home exercise program will parallel the three phases of the treatment protocol with the gradual addition of increasingly difficult exercises every four weeks. To standardize home PFM exercises, each participant will be given a PFM diary describing the home PFMT exercises, in which they will record their adherence to the home program. Finally, all participants will be asked to refrain from seeking other forms of treatment (such as medication or surgery) during the study.

Standardization of treatment The physiotherapists delivering the interventions will be extensively trained in standardized treatment protocols and rigorous procedures to conduct both individual and group PFMT. During the course of the study, all evaluators will regularly meet with the study team to ensure that protocol consistency is being maintained, and to discuss concerns that may arise.

3.3 Outcome measures

3.3.1 Primary outcome measure The primary outcome measure of this trial will be the percent reduction in the number of UI episodes one year post-randomization, as measured by a seven-day bladder diary.[70] For example, a participant experiencing four leaks per week pre-intervention and two leaks per week at the one-year follow-up would be considered to have had a 50% reduction in leakage episodes. The number of leakage episodes (as measured by the seven-day diary) is considered one of the most reliable measures of success for incontinence treatment and has been widely used in this type of research.[36,49,66,76] Moreover, the reduction in the number of daily leaks, expressed as a percentage, is information that can be easily understood by participants in pre-treatment counseling. As a measurement tool, the seven-day diary has a high compliance rate and good reproducibility.[51,73]

Secondary outcome measures Several secondary outcomes will be assessed in this study, in line with recommendations of the Fourth International Consultation on Incontinence[82] and the International Continence Society.[69]

1.The seven-day bladder diary: The number of micturition per day/night will be monitored to document urinary frequency during the day and nocturia.[51]
2. The 24-hour pad test: This is a validated measure of quantity of urine leakage in 24 hours, as measured by the weight of the pads used during a 24-hour period minus the weight of the pads before the test. It is identified as a realistic appraisal of typical urine loss during ordinary daily activities.[52] The upper limit of "normal" for the 24-hour pad test has been defined for continent women as 1.3 g.[76]
3. The International Consultation on Incontinence Questionnaire (ICIQ) Modules: Five ICIQ modules will be used, which provide brief and robust measures of UI symptoms, quality of life, and outcome of treatment.[55]
i. The ICIQ-Urinary Incontinence short form: A four-item questionnaire, which evaluates the impact of incontinence symptoms on quality of life and treatment outcome.[53]
ii. The ICIQ-Nocturia Module: A two-item questionnaire evaluating the impact of nocturia symptoms on quality of life and treatment outcome.[54]
iii. The ICIQ-Vaginal Symptoms Module: A 14-item questionnaire, which evaluates the impact of vaginal symptoms and associated sexual matters on quality of life and outcome of treatment.[56]
iv. The ICIQ-Gender-Specific Sexual Matters Module: a four-item questionnaire for evaluating sexual matters associated with female lower urinary tract symptoms.[57]
v. The ICIQ-Lower Urinary Tract Symptoms Quality of Life Module: A 20-item questionnaire, which evaluates quality of life in urinary incontinent patients.[55]4. The Geriatric Self-Efficacy Index: A 20-item questionnaire that enables measurement of a person's confidence in their ability to prevent urine loss. It has been shown to be a reliable and valid tool for older women with UI.[58]
5. The Broom Self-Efficacy Index (Part A): A 14-item questionnaire to evaluate women's confidence in performing PFM exercise.[59] The Broom self-efficacy index has solid psychometric properties and is a useful tool to measure self-efficacy for PFM exercises.[83]
6. Patient Global Impression of Improvement (PGI-I): A single-item global index used to measure improvement of urinary continence following PFMT on a seven-point scale that ranges from "very much better" to "very much worse." PGI-I has shown acceptable convergent and discriminant validity for measuring outcomes in studies on behavioral treatment for UI.[60,84]
7. Satisfaction with treatment: A single-item tool was used to document and capture perceived satisfaction with treatment: "satisfied" (does not need other treatments); "unsatisfied" (would like another treatment for UI).[37]
8. PFM strength, morphometry and function.
i. PFM strength on the Oxford Scale (digital palpation): The Oxford Scale is a five-point scale used by physiotherapists to assess PFM strength.[61,85]
ii. PFM function: An intravaginal dynamometric speculum, designed by members of this research team, will be used to measure passive (tone) and active forces (strength), speed of contraction, and coordination with cough and endurance [39]. This instrument has been widely assessed for its psychometric properties including its reliability, validity and responsiveness.[62,86]
iii. PFM morphometry: A Siemens Acuson Antares system with a 3–5-MHz curvilinear 3D/4D probe (in Montreal) and a GE Voluson Expert system with a 2–6-MHz curvilinear 3D/4D probe (in Sherbrooke) will be used to evaluate several morphometric parameters at rest, during PFM contraction and on effort (cough and Valsalva): levator hiatus area and diameter, bladder neck position and displacement as well as levator plate height, using a validated and reliable methodology.[87-89]

Costs related to interventions The health system perspective is retained in the economic analysis; consequently, only direct costs will be taken into account.[90] The *Dowell-Bryant Incontinence Cost Index (DBICI)* is a universally-applicable questionnaire that has been adapted to measure the intervention costs for the two groups.[64] The validated DBICI has been used in RCTs with community- dwelling populations of women age 40 and over for non-surgical UI interventions[91], and is recommended by the International Consultation on Incontinence Research Guidelines.[76] Section 1 of the DBICI documents includes monthly self-reported personal incontinence expenditures (disposable and reusable incontinence products) and is used integrality. Section 2 details treatment expenditures, and has been adapted as follows:
1) Treatment costs: rather than recording self-reported costs, treatment duration (hours) for each participant will be based on statistics maintained by the research coordinators using a standardized form developed for a previous cost-effectiveness study (Moffet, Tousignant and Nadeau, CIHR operating grant MCT-91011). The number of hours will be multiplied by the mean hourly salary of the physiotherapists participating in the study. For the group-based PFMT, the total number of hours will be divided by the number (n=8) of intended class participants (i.e., not actual attendance).

2) Other consultations: the frequency of visits to incontinence-related medical professionals will be documented by category (general practitioners and specialists). A research assistant will phone participants at three- and six-months post intervention in order to collect information. Estimated costs will be based on the frequency and the mean cost per visit based on the public health system regulations for each category (Régie de l'assurance maladie du Québec). Medication costs will be excluded from the trial, as patients with UI medications will be not be considered eligible for this study.

Socio-demographic data: Subjects will be queried on basic socio-demographic data including age, BMI, general health status, medical history and medications, parity and obstetric history, as well as duration of UI and current pad use.

Adherence: Participants will be provided with diaries to record home exercise adherence for the 12-week treatment session. In addition, exercise maintenance will be assessed with a standardized questionnaire, at three- and six-months post intervention, as well as at the 12-month follow-up evaluation. Additionally, adherence to each supervised weekly treatment session will be recorded by the physiotherapists

3.4 Methods for protecting against sources of bias

- Blinding subjects to their treatment assignment is not possible. However, to eliminate observer bias, all research members who are responsible for outcome assessments (PI, physiotherapist-evaluators) will be blinded to group allocations. Participants will also be asked not to disclose their group allocation and will be unaware of details of the other physiotherapy intervention.
- Ten evaluating physiotherapists (five in Montreal & five in Sherbrooke) will be trained to administer a standardized evaluation protocol. They will not be involved in treatment.
- The participants in each treatment group (individual and group-based) will be treated on different days and in different rooms, and have no direct contact with each other.
- To minimize differences due to seasonal variances in the women's participation levels and symptom exacerbation, individual and group-based sessions will both be conducted throughout the year.

3.5 Sample size and statistical power estimation

Primary hypothesis (main Outcome)

General approach and underlying assumptions: Sample size was calculated to ensure adequate power and type I error rate for testing the primary hypothesis of non-inferiority of the group-based vs. individual interventions in achieving the relative reduction (%) in the number of UI episodes at one year. Sample size calculations followed CONSORT guidelines for non-inferiority trials.[92] First, based on clinical relevance[93] (minimum clinically relevant difference = 10%) and pilot data, the 'margin of equivalence' (i.e. the upper limit of the non-inferiority interval) was set to a corresponding 10% difference between mean percent reduction in the number of UI episodes between individual minus group-based interventions, implying that the null hypothesis (H0) is H0:d<=10% where d denotes the true difference between the two interventions, while H1:d>10%. According to the CONSORT guidelines, this choice of the "margin of equivalence" implies that the non-inferiority hypothesis should be rejected whenever the upper bound of the two-tailed (1 - 2α)% confidence interval (CI) for the difference between the two mean percent reductions exceeds 10%, where (in the specific context of a non-inferiority trial) α is the selected risk of a false acceptance of the non-inferiority hypothesis (based on a one-tailed CI-based test, equivalent to an independent-group t-test); i.e., a false acceptance of a truly inferior intervention.[92] A conservative standard 95% CI was set, which corresponds to a stringent one-tailed $\alpha = 0.025$ ($2\alpha = 1-0.95 = 0.05$, hence $\alpha=0.025$). Similar to other recent non-inferiority trials[94,95] and CONSORT guidelines,[92] it was assumed that the true difference between the mean reduction (%) achieved by the two interventions will be zero (i.e., equally effective).[92] Note that this assumption of equivalence of the two interventions is consistent with both: (a) clinical expectations, and (b) (limited) published evidence in young and middle-aged women with UI.[96-98] Finally, two published trials evaluated similar interventions in older women reported within-group standard deviations (SD) of the individual percent reduction scores of about 27%[66,99] Accordingly, the SD will be set to 27% for calculations.

Sample size calculations: Under these assumptions, the estimated sample size will need to ensure high (90%) power to demonstrate non-inferiority of the group-based intervention (assuming, the true difference is 0%). Thus, N will be calculated so that the probability that the upper boundary of the two-tailed 95% CI for the difference in the mean reduction (individual – group) excludes the 'upper threshold of non-inferiority' (10% difference), and will reach at least 90%**.** The sample size calculations will be performed using the program in the PASS software package, designed specifically for power/sample size estimation for non-inferiority trials.[100] Under the assumptions outlined above, **155** subjects are needed per group, for a total of **310 subjects**. To account for possible loss to follow-up, a 15% attrition rate is expected at one year (in line with both pilot data and published trials).[36,37,42,49] Thus, an additional **54** participants will be recruited per group, increasing total participants to 364 (364*(1–0.15) = 310).

Power for testing secondary hypotheses Because secondary objective 2 will require participants to fill questionnaires and as such, impose longer study visit times, it will be necessary to confirm that the sample size calculated (to address the primary and secondary objective 1) provides adequate power to address secondary objective 2. The sample size of this study has been set to **155** subjects per group, for a total **N=310**, based on the power calculations for the primary hypothesis (see above). Accordingly, the power to demonstrate non-inferiority (based on a pre-specified threshold) was estimated while keeping the error rate of a false 'acceptance' of non-inferiority at 0.025 (for a one-tailed independent-group t-test based on a 95% CI - see section 3.7.1. above). Pilot data (section 1) or previous publications were used to derive the assumptions necessary for these calculations.[37,101] Adequate to excellent power has been established to address objective 2 (**79.3%** and 91.4%). Formal power analysis was not calculated for objective 3 (PFM function), as this analysis is **only exploratory in nature.**

3.6 Planned recruitment rate PFMT is a non-invasive intervention with no side effects, recognized for its positive impact on UI. As a result, women are usually willing to participate in such studies. Furthermore, Quebec, where this study will take place, currently has a shortage of physiotherapists, with only a few members of the association trained to provide PFMT. Waiting lists at hospitals and private clinics are at least six months long. Thus, a high response rate is anticipated, as well as the successful completion of the research protocol. As recruitment will be conducted over four years, 364 subjects will need to be recruited over 48 months to achieve the required sample size in a timely manner. It will be necessary to recruit at least 7.6 subjects per month through collaborations (7 clinics X 1.1 subjects = 7.6 x 48 months = 364) to achieve the required sample size. Based on past experience, this is deemed to be achievable.

3.7 Loss to follow up Based on the results of this research team's previous RCT, and cohort and feasibility studies, no significant problem is expected with treatment adherence in either group. Participants (n=27) in the previous feasibility study complied with study demands in terms of attendance at the treatment sessions (90%), and completed the daily exercise program (78%) and data collection (95%).[49] Post-study focus groups identified "*close supervision by the physiotherapist"* and a *"short daily PFM exercise program"* as key facilitators in the participant's completion of a 12-week group-based PFMT program and adherence to the daily home PFM exercise program.[42] Furthermore, participants in this research team's cohort study (n =174) complied with treatment and evaluation sessions (90%).[50,81]

Findings from previous studies will be used to promote treatment adherence [42,49,81] as follows Individual consultations using vaginal digital palpation will ensure participants can perform a PFM contraction correctly. The physiotherapist will be available prior to and after individual and group treatment sessions to provide tips and answer questions regarding the weekly treatment and home exercise program. An exercise diary will be given to each participant to bring home and will be verified by the physiotherapist each week throughout the treatment. The home exercise program will be comprised of a short and simple daily PFM exercise program. Women in both groups will receive a schedule for their two post-treatment follow-up evaluations. Follow-up contact calls will be made at six-, nine- and 12-months post-randomization to maintain contact and to minimize loss to follow-up. Evaluators will solicit information from participants on their intention to change address, and on the frequency of visits to incontinence-related medical professionals. They will also verify if medications have been prescribed, surgery conducted, and if there are any other health problems likely to influence UI. Participates will be reimbursed for their travel and parking expenses related to participation in the study.

4. STATISTICAL ANALYSES

4.1. General analytical strategy As opposed to conventional superiority trials, in non-inferiority trials *per-protocol analysis* is generally preferable to *intention-to-treat* (ITT) analysis[92] because the postulated hypothesis being tested assumes no difference between the two interventions.[92] Moreover, the ITT approach includes all subjects who were initially randomized, regardless of their adherence. Thus, they may dilute the true difference between the interventions in the case of non-adherence or loss to follow-up. [92] In an extreme case, if most subjects do not adhere to the assigned intervention or fail to complete the follow-up assessment, the ITT analysis will automatically show no difference. To prevent such paradoxes, main analysis will rely on the *'per-protocol'* approach; i.e. include only participants who complete the one-year assessment. On the other hand, because of its popularity, the ITT approach will be used in 'sensitivity analyses', as recommended by the CONSORT guidelines for non-inferiority trials.[92] Furthermore, analyses will focus on outcomes (primary or secondary) at one year (additional repeated-measure analyses are outlined below). Because all primary and secondary outcomes are measured on continuous scales (section 3.4), the primary outcome analysis method outlined below will also apply to secondary outcomes. However, the analyses of secondary outcomes will be exploratory in nature and some outcomes (e.g. PFM function) may have limited statistical power.

Preliminary descriptive analyses will compare the subjects in the two trial arms using mean, median, standard deviation and inter-quartile range (IQR) for continuous and frequency distributions for categorical and binary variables. Any variable for

which the difference between the two arms is considered clinically relevant will be adjusted for multivariable analyses (see below). The distribution of the outcome scores will be assessed for normality of residuals using the Shapiro-Wilk test. In the case of significant violation of the normality, appropriate parametric (e.g. logarithmic or Box-Cox[102]) or non-parametric[103] transformations will be applied to meet the assumptions underlying univariate t-tests, multivariable linear regressions and mixed linear models analyses (see below).

4.2 Analyses for the primary outcome at one year (and secondary outcomes) The primary analysis will test the main hypothesis that group-based PFMT is not inferior to individual treatment with respect to the primary outcome of relative reduction (%) in the number of leakage episodes one year post-randomization (for all outcomes, the analyses outlined below will be repeated using data in post-intervention assessment). As recommended for non-inferiority trials, the hypothesis of non-inferiority will only be accepted if the upper bound of the two-tailed (1-2α) 95% CI (corresponding to a conservative Type I error of 0.025 for the one-tailed independent-group t-test) for the mean difference (in percent reduction) excludes the non-inferiority threshold,[92] set to 10% difference. This basic analysis will be extended to multivariable analyses. Specifically, two multivariable linear models of increasing complexity will be used to adjust the estimated difference between the percent reduction in the two groups for, respectively: (1) only the two stratification variables (center and type of UI), as well as the baseline number of UI episodes (to account for regression to the mean phenomenon), and (2) (if necessary) in addition to variables in models (i): any variable for which a clinically important imbalance between the two arms is revealed by descriptive analyses (see above). In both models, two-way interactions between the trial arm and (a) center, and (b) UI type, will be tested using one-df model-based F-tests to verify if the difference between the effects of the two interventions depends on these stratification variables. In the case of a statistically significant interaction ($p<0.05$ for the F-test), the intervention effects will be estimated and tested separately in the respective groups. The results of multivariable linear regression analyses will be summarized in terms of adjusted mean difference in the outcomes in the two trial arms, together with 95% model-based CI's. Furthermore, to the analyses for primary and secondary outcomes at one year (section 4.2), a sensitivity ITT analysis will be conducted.

4.3 Economic analysis Mean, median and interquartile (semi IQ) will be used to describe the costs. Cost-effectiveness analysis will be performed on the main outcome: reduction in number of leakage episodes (%) one year after randomization and expressed as a ratio of incremental costs and percentage of leakages. In order to verify the robustness of the economic analysis, a sensitivity analysis will be conducted on the hypothesis that the costs for each subject should be between the 25th and 75th percentiles of the cost distribution**.**

4.4 Multivariable models to predict response to treatment Multivariable logistic regression analyses will be employed to assess patient characteristics associated with an increased likelihood of response to treatment, defined as at least 50% reduction in the number of UI episodes at one year**.**

5. TRIAL MANAGEMENT The PI and two research coordinators (one located in Sherbrooke and the other in Montreal) will be visiting each participating urology and gynecology clinic in order to promote and monitor progress in recruitment. The PI, evaluators, physiotherapists and the Sherbrooke and Montreal coordinators will also conduct conference calls in order to monitor the study's progress. Other members of the team will be kept informed of progress through a newsletter every six months. All collected data will be anonymous and kept under lock and key at Dr Dumoulin's laboratory at the Institut universitaire de gériatrie de Montréal. Data collected in Sherbrooke will also be anonymous and kept under lock and key in Dr Morin's office. Data will be entered weekly into a computerized database system ***SPSS data Entry 4.0***. Every week, the project coordinators will review all original questionnaires and forms and compare them to the print copies of the entered data to ensure consistency. Any identified errors will result in corrections to the study database. The database will be backed up on a weekly basis. A PIN number known only to the research group will be required to access the data entry computer. A final quality control step will be taken at the time of the data analysis by the trial statistician. Frequency distributions and ranges will be analyzed to detect outliers that could signal potential errors. The data will be analyzed without any nominative identifiers.

6. IMPACT If group-based PFMT classes prove to be equally effective and more cost-effective than individual one-on-one PFMT sessions, this trial will have a positive impact on the accessibility of continence care in Canada. This information will be relevant to clinicians, clinical decision-makers and administrative stakeholders. It will have implications for organizations and the administration of continence care services. **If implemented as a first-line treatment, group-based PFMT will reduce the cost of treatment per individual and the prevalence and medical complications of UI in older women.** The results of this study will be disseminated through national and international scientific and professional conferences, in addition to undergraduate and post-graduate courses in PFM rehabilitation for physiotherapists. Once published, these results will provide clinicians with a new low-cost PFMT program to treat UI in older women.

7. TRIAL TEAM DETAILS Applicant and co-applicants: This project brings together dynamic individuals, including new and experienced researchers, from two Quebec universities: Université de Sherbrooke and Université de Montréal. This research team encompasses the disciplines of rehabilitation, gynecology, urology, research methodology, geriatrics and biostatistics. Each team member has specific and varied expertise in either UI, aging, women's health, RCTs or PFMT. **C. Dumoulin,** a physiotherapist by profession, has clinical and research experience in PFMT and dynamometry. She will be responsible for overseeing the project. She has conducted many studies on either group or individual PFMT (CIHR: MOP 84529; FRSQ: 14417). She previously conducted a CIHR-funded RCT (CIHR grant No.36053) entitled ***Pelvic floor muscle training for post-natal UI;*** and she recently completed a seven-year follow-up for this important RCT and another large cohort study on PFM-exercise-class success predictors. She is the PI and will be responsible for coordinating the team; hiring and training a research assistant, the evaluators and physical therapists; overseeing subject recruitment efforts; analyzing the results; and preparing the final reports and publications. **MH. Mayrand,** a gynecologist and researcher at the Centre hospitalier universitaire de Montréal, has expertise in research design and RCT methodology in women's health. She contributed to the protocol development, power calculations, and the selection of the statistical analysis methodology. She will participate in recruitment and assist in the preparation of the manuscripts. **M. Morin,** a young researcher at the CRCEL, is also a physiotherapist by profession. She is currently the PI on a CIHR multi-site RCT examining a physiotherapy intervention for women with pelvic and perineal pain. She will be responsible for the Sherbrooke recruitment sites. She contributed to the study's protocol development and will assist in analyzing the findings and in the preparation of the manuscripts. **M. Tousignant** is a researcher at the Institut universitaire de gériatrie de Sherbrooke with extensive expertise in evaluating the cost-effectiveness of rehabilitation interventions in older adults (CIHR MCT-91011). He contributed to the protocol development, will be responsible for the cost-effectiveness evaluation and will assist in interpreting cost-related findings. **Michal Abrahamowickz** is a biostatistician with extensive experience in RCTs. He contributed extensively to sample size calculations and the choice of statistical analyses. He will oversee data analysis and assist in the interpretation of results.

The following clinicians have agreed to collaborate in the research project by promoting the study and assisting in the recruitment of participants: **J. Corcos, A. Girard**, **M. Jolivet, M-C. Lemieux**, **M.H. Mayrand, C. Tannenbaum, L.M. Tu**. **Participating centers:** seven urological or urogynecology clinics will be involved in recruitment.

References

1. Haylen BT, De Ridder D, Freeman RM, et al. An International Urogynecological Association (IUGA)/International Continence Society (ICS) joint report on the terminology for female pelvic floor dysfunction. *Neurourology and Urodynamics: Official Journal of the International Continence Society.* 2010;29(1):4-20.
2. Milsom I, Altman D, Lapitan M, Nelson R, Sillen U, Thom D. Epidemiology of urinary (UI) and faecal (FI) incontinence and pelvic organ prolapse (POP). *Incontinence. Fourth International Consultation on Incontinence. 4th Edition* France: Health Public Publication Ltd; 2009:35-111.
3. Hannestad YS, Rortveit G, Sandvik H, Hunskaar S. A community-based epidemiological survey of female urinary incontinence:: The Norwegian EPINCONT Study. *Journal of clinical epidemiology.* 2000;53(11):1150-1157.
4. Herschorn S, Corcos J, Gajewski J, Schulz J, Ciu E. Canadian urinary bladder survey: population-based study of symptoms and incontinence. *NEUROUROLOGY AND URODYNAMICS.* 2003;22(5):49-49.
5. Statistique Canada. Population projetée par groupe d'âge et sexe selon trois scénarios de projection au 1er juillet pour les années 2010, 2011, 2016, 2021, 2026,2031 et 2036. http://www40.statcan.gc.ca/l02/cst01/demo23e-fra.htm.
6. Resnick NM, Yalla SV, Laurino E. The pathophysiology of urinary incontinence among institutionalized elderly persons. *New England Journal of Medicine.* 1989;320(1):1-7.
7. Faltin D-L. Épidémiologie et définition de l'incontinence urinaire féminine. *Journal de gynécologie obstétrique et biologie de la reproduction.* 2009;38(8):S146-S152.
8. Hunskaar S, Vinsnes A. The quality of life in women with urinary incontinence as measured by the sickness impact profile. *Journal of the American Geriatrics Society.* 1991;39(4):378-382.
9. Johnson TM, Kincade JE, Bernard SL, Busby-Whitehead J, Hertz-Picciotto I, DeFriese GH. The association of urinary incontinence with poor self-rated health. *Journal of the American Geriatrics Society.* 1998;46(6):693-699.
10. Ko Y, Lin S-J, Salmon JW, Bron MS. The impact of urinary incontinence on quality of life of the elderly. *Am J Manag Care.* 2005;11(4 Suppl):S103-111.
11. Temml C, Haidinger G, Schmidbauer J, Schatzl G, Madersbacher S. Urinary incontinence in both sexes: prevalence rates and impact on quality of life and sexual life. *Neurourology and Urodynamics: Official Journal of the International Continence Society.* 2000;19(3):259-271.
12. Sen I, Onaran M, Aksakal N, et al. The impact of urinary incontinence on female sexual function. *Advances in therapy.* 2006;23(6):999-1008.
13. Borer KT. Physical activity in the prevention and amelioration of osteoporosis in women. *Sports medicine.* 2005;35(9):779-830.
14. Ravaglia G, Forti P, Lucicesare A, et al. Physical activity and dementia risk in the elderly: findings from a prospective Italian study. *Neurology.* 2008;70(19 Part 2):1786-1794.
15. Kokkinos P. Physical activity and cardiovascular disease prevention: current recommendations. *Angiology.* 2008;59(2_suppl):26S-29S.
16. Pines A. Lifestyle and diet in postmenopausal women. *Climacteric.* 2009;12(sup1):62-65.
17. Canadian Continence Foundation. Impacts of incontinence in Canada. Brieffing document for policy makers from the Canadia Continence Foundation. . May 2009.
18. DuBeau C, Kuchel G, Johnson II T, Palmer M, Wagg A. Incontinence in the frail elderly. *Incontinence. Fourth International Consultation on Incontinence. 4th Edition.* Vol 292010:165-178.
19. Fantl JA. *Urinary incontinence in adults: acute and chronic management.* Department of Health and Human Services; 1996.
20. Hay Smith J, Berghmans B, Burgio K, et al. Adult conservative management in Incontinence. *Incontinence. Fourth International Consultation on Incontinence. 4th Edition*. France: Health Public Publication Ltd; 2009:1025-1120.
21. Bélisle S, Blake J, Basson R, et al. Canadian consensus conference on menopause, 2006 update. *Journal of Obstetrics and Gynaecology Canada.* 2006;28(2):S7-S9.
22. Canadian Continence Foundation. Canadian Consensus Conference on Urinary Incontinence: clinical practice guidelines for adults. 2001; http://www.continence-fdn.ca.
23. Dumoulin C, Hay-Smith J. Pelvic floor muscle training versus no treatment, or inactive control treatments, for urinary incontinence in women. *status and date: Edited (no change to conclusions), published in.* 2010(1).
24. Imamura M, Abrams P, Bain C, et al. Systematic review and economic modelling of the effectiveness and cost-effectiveness of non-surgical treatments for women with stress urinary incontinence. *Health Technology Assessment.* 2010.
25. Burns PA, Pranikoff K, Nochajski TH, Hadley EC, Levy KJ, Ory MG. A comparison of effectiveness of biofeedback and pelvic muscle exercise treatment of stress incontinence in older community-dwelling women. *Journal of Gerontology.* 1993;48(4):M167-M174.
26. Wells TJ, Brink CA, Diokno AC, PhD RW, Gillis GL. Pelvic muscle exercise for stress urinary incontinence in elderly women. *Journal of the American Geriatrics Society.* 1991;39(8):785-791.

27. Miller JM, Ashton-Miller JA, DeLancey JO. A pelvic muscle precontraction can reduce cough-related urine loss in selected women with mild SUI. *Journal of the American Geriatrics Society.* 1998;46(7):870-874.
28. Dougherty MC, Dwyer JW, Pendergast JF, et al. A randomized trial of behavioral management for continence with older rural women¶. *Research in nursing & health.* 2002;25(1):3-13.
29. Spruijt J, Vierhout M, Verstraeten R, Janssens J, Burger C. Vaginal electrical stimulation of the pelvic floor: a randomized feasibility study in urinary incontinent elderly women. *Acta obstetricia et gynecologica Scandinavica.* 2003;82(11):1043-1048.
30. Swanson JG, Skelly J, Hutchison B, Kaczorowski J. Urinary incontinence in Canada. National survey of family physicians' knowledge, attitudes, and practices. *Canadian family physician.* 2002;48(1):86-92.
31. Bland DR, Dugan E, Cohen SJ, et al. The effects of implementation of the Agency for Health Care Policy and Research urinary incontinence guidelines in primary care practices. *Journal of the American Geriatrics Society.* 2003;51(7):979-984.
32. II TJ, Kincade J, Bernard S, Busby-Whitehead J, DeFriese G. Self-care practices used by older men and women to manage urinary incontinence: results from the national follow-up survey on self-care and aging. *Journal of the American Geriatrics Society.* 2000;48(8):894-902.
33. St John W, James H, McKenzie S. “Oh, that's a bit of a nuisance”: Community-dwelling clients' perspectives of urinary continence health service provision. *Journal of WOCN.* 2002;29(6):312-319.
34. Dugan E, Roberts CP, Cohen SJ, et al. Why older Community-Dwelling adults do not discuss urinary incontinence with their primary care physicians. *Journal of the American Geriatrics Society.* 2001;49(4):462-465.
35. Walker JM. Curricular content on urinary incontinence in entry-level physical therapy programmes in three countries. *Physiotherapy Research International.* 1998;3(2):123-134.
36. Kim H, Yoshida H, Suzuki T. The effects of multidimensional exercise treatment on community-dwelling elderly Japanese women with stress, urge, and mixed urinary incontinence: a randomized controlled trial. *International journal of nursing studies.* 2011;48(10):1165-1172.
37. Sherburn M, Bird M, Carey M, Bø K, Galea MP. Incontinence improves in older women after intensive pelvic floor muscle training: An assessor-blinded randomized controlled trial. *Neurourology and urodynamics.* 2011;30(3):317-324.
38. Borghuis M. Group remedial therapy in general practice for women with incontinence symptoms. *Physiotherapy.* 1994;87(5):235-241.
39. Demain S, Smith JF, Hiller L, Dziedzic K. Comparison of group and individual physiotherapy for female urinary incontinence in primary care: pilot study. *Physiotherapy.* 2001;87(5):235-242.
40. Cook T. Group treatment of female urinary incontinence: literature review. *Physiotherapy.* 2001;87(5):226-234.
41. Ewles L, Simnett I. *Promoting health: a practical guide.* Baillière Tindall London; 1999.
42. Martin C, Dumoulin C. Pelvic floor exercise classes for Urinary incontinence in older women: a feasibility study. Presentation *World Congress Physical Therapy, Vancouver, Canada.* June 2007.
43. Kääpä EH, Frantsi K, Sarna S, Malmivaara A. Multidisciplinary group rehabilitation versus individual physiotherapy for chronic nonspecific low back pain: a randomized trial. *Spine.* 2006;31(4):371-376.
44. Miller-Kovach K, Hermann M, Winik M. The psychological ramifications of weight management. *Journal of women's health & gender-based medicine.* 1999;8(4):477-482.
45. Elliot V, Dumoulin C, Morin M, Bourbonnais D, Lemieux M, Gauthier R. Physical Therapy for Persistent Postpartum Stress Urinary Incontinence: a Seven Year Follow-up Study, Oral presentation. *International Continence Society Annual Congress, San Francisco, US.* Sept. 2009.
46. Alewijnse D, Mesters I, Metsemakers J, Van Den Borne B. Predictors of long-term adherence to pelvic floor muscle exercise therapy among women with urinary incontinence. *Health education research.* 2003;18(5):511-524.
47. Dumoulin C, Lemieux M-C, Bourbonnais D, Gravel D, Bravo G, Morin M. Physiotherapy for persistent postnatal stress urinary incontinence: a randomized controlled trial. *Obstetrics & Gynecology.* 2004;104(3):504-510.
48. Dumoulin C, Bourbonnais D, Morin M, Gravel D, Lemieux M-C. Predictors of success for physiotherapy treatment in women with persistent postpartum stress urinary incontinence. *Archives of physical medicine and rehabilitation.* 2010;91(7):1059-1063.
49. Dumoulin C, Corriveau C, Corcos C, Tannebaum C, Tu L. Feasibility study examining physiotherapy exercise classes for older women with urinary incontinence. Poster presentation. *34th Annual Meeting of the International Urogynecological Association, Come Lake, Italy.* June 2009.
50. Dumoulin C, Boudreault N, Corriveau H, Corcos C, Tannenbaum C, Lemieux M. Characterizing and comparing pelvic floor muscle function in continent and urinary incontinent older women using dynamometry. Poster presentation. *Joint ICS-IUGA Annual Meeting Abstract, Toronto, Canada.* Aug. 2010.
51. Locher JL, Goode PS, Roth DL, Worrell RL, Burgio KL. Reliability assessment of the bladder diary for urinary incontinence in older women. *The Journals of Gerontology Series A: Biological Sciences and Medical Sciences.* 2001;56(1):M32-M35.

52. Karantanis E, Allen W, Stevermuer T, Simons A, O'Sullivan R, Moore K. The repeatability of the 24-hour pad test. *Int Urogynecol J.* 2005;16(1):63-68.
53. Avery K, Donovan J, Peters TJ, Shaw C, Gotoh M, Abrams P. ICIQ: a brief and robust measure for evaluating the symptoms and impact of urinary incontinence. *Neurourology and urodynamics.* 2004;23(4):322-330.
54. Abraham L, Hareendran A, Mills IW, et al. Development and validation of a quality-of-life measure for men with nocturia. *Urology.* 2004;63(3):481-486.
55. Kelleher C, Cardozo L, Khullar V, Salvatore S. A new questionnaire to assess the quality of life of urinary incontinent women. *BJOG: An International Journal of Obstetrics & Gynaecology.* 1997;104(12):1374-1379.
56. Price N, Jackson S, Avery K, Brookes S, Abrams P. Development and psychometric evaluation of the ICIQ Vaginal Symptoms Questionnaire: the ICIQ-VS. *BJOG: An International Journal of Obstetrics & Gynaecology.* 2006;113(6):700-712.
57. Jackson S, Donovan J, Brookes S, Eckford S, Swithinbank L, Abrams P. The Bristol female lower urinary tract symptoms questionnaire: development and psychometric testing. *BJU International.* 1996;77(6):805-812.
58. Tannenbaum C, Brouillette J, Korner-Bitensky N, et al. Creation and testing of the geriatric self-efficacy index for urinary incontinence. *Journal of the American Geriatrics Society.* 2008;56(3):542-547.
59. Broome B. Development and testing of a scale to measure self-efficacy for pelvic muscle exercises in women with urinary incontinence. 1998.
60. Yalcin I, Bump RC. Validation of two global impression questionnaires for incontinence. *American journal of obstetrics and gynecology.* 2003;189(1):98-101.
61. Laycock J. Assessment and treatment of pelvic floor dysfunction. *Physiotherapy.* 1992;78(10):737.
62. Dumoulin C, Bourbonnais D, Lemieux MC. Development of a dynamometer for measuring the isometric force of the pelvic floor musculature. *Neurourology and Urodynamics: Official Journal of the International Continence Society.* 2003;22(7):648-653.
63. Brækken IH, Majida M, Ellstrøm-Engh M, Dietz HP, Umek W, Bø K. Test–retest and intra-observer repeatability of two-, three-and four-dimensional perineal ultrasound of pelvic floor muscle anatomy and function. *Int Urogynecol J.* 2008;19(2):227-235.
64. Dowell C, Bryant C, Moore K, Simons A. Calculating the direct costs of urinary incontinence: a new test instrument. *Bju International.* 1999;83(6):596-606.
65. Ouslander JG, Hepps K, Raz S, Su HL. Genitourinary dysfunction in a geriatric outpatient population. *Journal of the American Geriatrics Society.* 1986;34(7):507-514.
66. Burgio KL, Goode PS, Locher JL, et al. Behavioral training with and without biofeedback in the treatment of urge incontinence in older women: a randomized controlled trial. *Jama.* 2002;288(18):2293-2299.
67. Tannenbaum C, Bachand G, Dubeau CE, Kuchel GA. Experience of an incontinence clinic for older women: no apparent age limit for potential physical and psychological benefits. *Journal of women's health & gender-based medicine.* 2001;10(8):751-756.
68. Bradley CS, Rahn DD, Nygaard IE, et al. The questionnaire for urinary incontinence diagnosis (QUID): validity and responsiveness to change in women undergoing non-surgical therapies for treatment of stress predominant urinary incontinence. *Neurourology and urodynamics.* 2010;29(5):727-734.
69. Hilton P, Robinson D. Defining cure. *Neurourology and urodynamics.* 2011;30(5):741-745.
70. Lentz G, Stanton S. Urinary diary: designing a shorter diary. *Int Urogynecol J.* 1992;3(3):69.
71. Whitehead W. Report of an international workshop on management of constipation. *Gastroenterol Int.* 1991;4:99-113.
72. World Health Organization. Physical status: The use of and interpretation of anthropometry, Report of a WHO Expert Committee. 1995.
73. Bump R, Mattiasson A, Brubaker L. The standardization of terminology of female organ prolapse and pelvic floor dysfunction. *Am J Obstet Gynecol.*18.
74. Folstein MF, Folstein SE, McHugh PR. "Mini-mental state": a practical method for grading the cognitive state of patients for the clinician. *Journal of psychiatric research.* 1975;12(3):189-198.
75. Anderson K, Chapple C, Cardozo L, Crutz F. Pharmacological treatment of urinary incontinence. In: Abrams P, Cardozo L, Khoury S, Wein A, eds. *Incontinence. Fourth International Consultation on Incontinence. 4th Edition. 2009*. France: Health Public Publication Ltd; 2009:631-699.
76. Staskin D, Kelleher C, Avery K, et al. Initial assessment of urinary and faecal incontinence in adult male and female patients. In: Abrams P, Cardozo L, Khoury S, Wein A, eds. *Incontinence: 4th International Consultation on Incontinence.* . Paris, France: Health Publications Ltd; 2009:311-412.
77. DiNubile N. Strength training. *Clinics in sports medicine.* 1991;10(1):33-62.
78. Gunnarsson M, Teleman P, Mattiasson A, Lidfeldt J, Nerbrand C, Samsioe G. Effects of Pelvic Floor Exercises in Middle Aged Women with a History of Naïve Urinary Incontinence:: A Population Based Study. *European urology.* 2002;41(5):556-561.
79. Dumoulin C, Chiva-Razavi S, Corriveau H. Is there a relationship between balance or lower extremity strength and

the impact of mixed urinary incontinence in aging community-dwelling women? Oral presentation. *International Continence Society Annual Congress, San Francisco, US.* Sept. 2009.
80. Bø K, Talseth T, Holme I. Single blind, randomised controlled trial of pelvic floor exercises, electrical stimulation, vaginal cones, and no treatment in management of genuine stress incontinence in women. *Bmj.* 1999;318(7182):487-493.
81. Madill S, Tang A, Pontbriand-Drolet S, Dumoulin C. Pelvic floor exercise classes for urinary incontinence in older women: How do they work, Poster presentation. *International Continence Society Congress. Glasgow, Scottland.* 2011.
82. Abrams P, Cardozo L, Khoury S, Wein A. *Incontinence, 4th.* Paris, France: Health Public Publication Ltd; 2009.
83. Chen S-Y. The development and testing of the pelvic floor muscle exercise self-efficacy scale. *The journal of nursing research: JNR.* 2004;12(4):257-266.
84. Srikrishna S, Robinson D, Cardozo L. Validation of the Patient Global Impression of Improvement (PGI-I) for urogenital prolapse. *Int Urogynecol J.* 2010;21(5):523-528.
85. Laycock J, Jerwood D. Pelvic floor muscle assessment: the PERFECT scheme. *Physiotherapy.* 2001;87(12):631-642.
86. Morin M, Dumoulin C, Gravel D, Bourbonnais D, Lemieux MC. Reliability of speed of contraction and endurance dynamometric measurements of the pelvic floor musculature in stress incontinent parous women. *Neurourology and urodynamics.* 2007;26(3):397-403.
87. Brauer M, Ter Kuile MM, Laan E, Trimbos B. Cognitive-affective correlates and predictors of superficial dyspareunia. *Journal of Sex & Marital Therapy.* 2008;35(1):1-24.
88. Dietz H. Ultrasound imaging of the pelvic floor. Part I: two-dimensional aspects. *Ultrasound in Obstetrics and Gynecology.* 2004;23(1):80-92.
89. Dietz H. Ultrasound imaging of the pelvic floor. Part II: three-dimensional or volume imaging. *Ultrasound in Obstetrics and Gynecology: The Official Journal of the International Society of Ultrasound in Obstetrics and Gynecology.* 2004;23(6):615-625.
90. Drummond MF, Sculpher MJ, Claxton K, Stoddart GL, Torrance GW. *Methods for the economic evaluation of health care programmes.* Oxford university press; 2015.
91. Simons A, Dowell C, Bryant C, Prashar S, Moore K. Use of the Dowell Bryant Incontinence Cost Index as a post-treatment outcome measure after non-surgical therapy. *Neurourology and Urodynamics: Official Journal of the International Continence Society.* 2001;20(1):85-93.
92. Piaggio G, Elbourne DR, Altman DG, Pocock SJ, Evans SJ, Group C. Reporting of noninferiority and equivalence randomized trials: an extension of the CONSORT statement. *Jama.* 2006;295(10):1152-1160.
93. Yalcin I, Peng G, Viktrup L, Bump RC. Reductions in stress urinary incontinence episodes: what is clinically important for women? *Neurourology and Urodynamics: Official Journal of the International Continence Society.* 2010;29(3):344-347.
94. CATT Research Group. Ranibizumab and bevacizumab for neovascular age-related macular degeneration. *New England Journal of Medicine.* 2011;364(20):1897-1908.
95. Le Henanff A, Giraudeau B, Baron G, Ravaud P. Quality of reporting of noninferiority and equivalence randomized trials. *Jama.* 2006;295(10):1147-1151.
96. de Oliveira Camargo F, Rodrigues AM, Arruda RM, Sartori MGF, Girão MJBC, Castro RA. Pelvic floor muscle training in female stress urinary incontinence: comparison between group training and individual treatment using PERFECT assessment scheme. *Int Urogynecol J.* 2009;20(12):1455-1462.
97. Lamb S, Pepper J, Lall R, et al. Group treatments for sensitive health care problems: a randomised controlled trial of group versus individual physiotherapy sessions for female urinary incontinence. *BMC women's health.* 2009;9(1):26.
98. Janssen C, Lagro-Janssen A, Felling A. The effects of physiotherapy for female urinary incontinence: individual compared with group treatment. *BJU international.* 2001;87(3):201-206.
99. Goode PS, Burgio KL, Locher JL, et al. Effect of behavioral training with or without pelvic floor electrical stimulation on stress incontinence in women: a randomized controlled trial. *Jama.* 2003;290(3):345-352.
100. Hintze J. PASS. Kaysville: NCSS, LLC. 2008; https://www.ncss.com/.
101. Borello-France DF, Downey PA, Zyczynski HM, Rause CR. Continence and quality-of-life outcomes 6 months following an intensive pelvic-floor muscle exercise program for female stress urinary incontinence: a randomized trial comparing low-and high-frequency maintenance exercise. *Physical therapy.* 2008;88(12):1545-1553.
102. Box GE, Cox DR. An analysis of transformations. *Journal of the Royal Statistical Society: Series B (Methodological).* 1964;26(2):211-243.
103. Ramsay JO. Monotone regression splines in action. *Statistical science.* 1988;3(4):425-441.
