# Supplementary material for: Group-Based vs Individual Pelvic Floor Muscle Training to Treat Urinary Incontinence in Older Women: A Randomized Clinical Trial
Source: JAMA Intern Med. 2020 Aug 3;180(10):1284–93. doi: 10.1001/jamainternmed.2020.2993 (PMC7400216; doi:10.1001/jamainternmed.2020.2993)
Supplement: Supplement 2. — eTable 1. Consensus of Exercise Reporting Template (CERT) eTable 2. Supervised Pelvic Floor Muscle Training Program eTable 3. Baseline Characteristics of the Population That Completed the Study vs the Population That Did Not Complete the Study eTable 4. Mean Values for the Primary and Urinary Incontinence Specific Secondary Outcomes in Individual and Group-Based Pelvic Floor Muscle Training at 12-Weeks and One-Year Follow-up eTable 5. Mean Values for the Impact On Other Lower Urinary Tract Symptoms, Vaginal, Sexual Symptoms and Self-Efficacy in Individual and Group-Based Pelvic Floor Muscle Training at 12-Weeks and One-Year Follow-up eTable 6. Primary Outcome- Percentage Reduction of Leakage Episodes in Individual and Group-Based Interventions at One-Year Follow-up According to Subgroups of Interest eTable 7. Odds Ratios for the Association Between the Intervention Arm (Group-Based Pelvic Floor Muscle Training vs Individual Pelvic Floor Muscle Training) and the Binary Outcome of Percentage Reduction of Leakage Episodes eTable 8. Minimal Clinically Important Differences at the One-Year Follow-up eTable 9. Other Treatments Taken During the Study Period eReferences [file jamainternmed-e202993-s002.pdf]

Supplementary Online Content

Dumoulin C, Morin M, Danieli C, et al; for the Urinary Incontinence and Aging Study Group. Group-based vs individual pelvic floor muscle training to treat urinary incontinence in older women: a randomized clinical trial. *JAMA Intern Med*. Published online August 3, 2020. doi:10.1001/jamainternmed.2020.2983

eTable 1. Consensus of Exercise Reporting Template (CERT)

eTable 2. Supervised Pelvic Floor Muscle Training Program

eTable 3. Baseline Characteristics of the Population That Completed the Study vs the Population That Did Not Complete the Study

eTable 4. Mean Values for the Primary and Urinary Incontinence Specific Secondary Outcomes in Individual and Group-Based Pelvic Floor Muscle Training at 12-Weeks and One-Year Follow-up

eTable 5. Mean Values for the Impact On Other Lower Urinary Tract Symptoms, Vaginal, Sexual Symptoms and Self-Efficacy in Individual and Group-Based Pelvic Floor Muscle Training at 12-Weeks and One-Year Follow-up

eTable 6. Primary Outcome- Percentage Reduction of Leakage Episodes in Individual and Group-Based Interventions at One-Year Follow-up According to Subgroups of Interest

eTable 7. Odds Ratios for the Association Between the Intervention Arm (Group-Based Pelvic Floor Muscle Training vs Individual Pelvic Floor Muscle Training) and the Binary Outcome of Percentage Reduction of Leakage Episodes

eTable 8. Minimal Clinically Important Differences at the One-Year Follow-up

eTable 9. Other Treatments Taken During the Study Period

eReferences

This supplementary material has been provided by the authors to give readers additional information about their work.

eTable 1. Consensus of Exercise Reporting Template (CERT)

Item	Description	Individual PFMT	Group-based PFMT
**1**	Detailed description of exercise equipment (e.g. weights, treadmill, ergometer etc.)	Treatment bed, chair, parallel bar, virtual reality game and screen, EMG biofeedback intra-vaginal probe	floor mats, chairs, parallel bars, virtual reality game, and screen
**2**	Detailed description of instructor expertise, qualifications, and/or training	Expertise: physiotherapist specialized in pelvic floor rehabilitation<br>Qualification: postgraduate program in PFM rehabilitation at Université de Montréal<br>Protocol training: 4-h workshop, written treatment protocol with checklist, individual supervision for three to six treatments.<br>Routinely, during the course of the study, all physiotherapists met with the study team to ensure consistency in the protocol and to discuss related concerns that may arise.	Expertise: physiotherapist specialized in pelvic floor rehabilitation<br>Qualification: postgraduate program in PFM rehabilitation at Université de Montréal<br>Protocol training: 4-h workshop, written treatment protocol with checklist, individual supervision for three to six treatments.<br>Routinely, during the course of the study, all physiotherapists met with the study team to ensure consistency in the protocol and to discuss related concerns that may arise.
**3**	Describe whether exercises are performed individually or in a group	Exercises were performed individually	Exercises were performed in groups of 6-8 women
**4**	Describe whether exercises are supervised or unsupervised; how they are delivered	Exercises were supervised by a trained physiotherapist	Exercises were supervised by a trained physiotherapist
**5**	Detailed description of how adherence to exercise is measured and reported	Adherence to each supervised weekly treatment session was recorded by the physiotherapist.<br>Adherence to home exercise:<br>- During the 12-week treatment was provided through an exercise diary filled by the participants and checked by the physiotherapists, every week.	Adherence to each supervised weekly treatment session was recorded by the physiotherapist.<br>Adherence to home exercise:<br>- During the 12-week treatment was provided through an exercise diary filled by the participants and checked by the physiotherapists, every week.

		- Exercise maintenance was assessed with a standardized questionnaire, 6, 9 and 12 months after the baseline assessment.<br>Exercise diary and maintenance questionnaires included the number of completed contractions per set, sets per day and days per week for each of the 4 PFM exercises (strengthening, endurance, coordination and rapidity).	- Exercise maintenance was assessed with a standardized questionnaire, 6, 9 and 12 months after the baseline assessment.<br>Exercise diary and maintenance questionnaires included the number of completed contractions per set, sets per day and days per week for each of the 4 PFM exercises (strengthening, endurance, coordination and rapidity).
**6**	Detailed description of motivation strategies	Motivational prompts were included in the exercise diary.<br>At the beginning of each treatment session, women reported their adherence to home exercise and tips and advice were given by the physiotherapist to improve adherence.<br>One education session (15 minutes) discussed motivation, exercise adherence, exercise maintenance and strategies to adopt in case of relapse.<br>Six- and nine-month follow-up phone calls were also used to reinforce strategies to improve adherence to home exercises.	Motivational prompts were included in the exercise diary.<br>At the beginning of each treatment session, women reported their adherence to home exercise and tips and advice were given by the physiotherapist to improve adherence.<br>One education session (15 minutes) discussed motivation, exercise adherence, exercise maintenance and strategies to adopt in case of relapse.<br>Six- and nine-month follow-up phone calls were also used to reinforce strategies to improve adherence to home exercises.
**7a**	Detailed description of decision rule(s) for determining exercise progression	The treatment progression was standardized.	The treatment progression was standardized.
**7b**	Detailed description of how exercise program was progressed	The program included three phases of gradual exercise progression; that is, the gradual addition of increasingly difficult exercises in terms of duration, repetition, and body position. Each phase lasted four weeks.	The program included three phases of gradual exercise progression; that is, the gradual addition of increasingly difficult exercises in terms of duration, repetition, and body position. Each phase lasted four weeks.
**8**	Detailed description of each exercise to enable replication	See eTable 2 for details of each weekly session of the 12-week PFM physiotherapy program	See eTable 2 for details of each weekly session of the 12-week PFM physiotherapy program
**9**	Detailed description of any home program component	Four PFM exercises (same as in the PFM physiotherapy program): knack (coordination), maximal contraction (strengthening), fast contractions (rapidity) and podiums (endurance).	Four PFM exercises (same as in the PFM physiotherapy program): knack (coordination), maximal contraction (strengthening), fast contractions (rapidity) and podiums (endurance).

		Five days a week, for the duration of the 12 week/treatment and 3 days a week, until 1-year follow-up.<br><br>The home program followed the treatment protocol progression. It was divided into three phases allowing for gradual exercise progression and one phase allowing maintenance; that is, the gradual addition of increasingly difficult exercises in terms of duration, repetition and position. Each of the three first phases lasted four weeks. The last phase lasted from the end of treatment to the one-year follow up. The complete content of the home exercise program was published previously.[1]	Five days a week, for the duration of the 12 week/treatment and 3 days a week, until 1-year follow-up.<br><br>The home program followed the treatment protocol progression. It was divided into three phases allowing for gradual exercise progression and one phase allowing maintenance; that is, the gradual addition of increasingly difficult exercises in terms of duration, repetition and position. Each of the three first phases lasted four weeks. The last phase lasted from the end of treatment to the one-year follow up. The complete content of the home exercise program was published previously.[1]
**10**	Describe any non-exercise components e.g. education, cognitive behavioural therapy etc	10-15-minute educational component present in every weekly session covering the anatomy and various functions of the PFMs; normal bladder control; voiding parameters; bladder irritants; fluid intake; toilet positions; and voiding dynamics. The complete content of this educational material was published previously.[1]	10-15-minute educational component present in every weekly session covering the anatomy and various functions of the PFMs; normal bladder control; voiding parameters; bladder irritants; fluid intake; toilet positions; and voiding dynamics. The complete content of this educational material was published previously.[1]
**11**	Describe the type and number of adverse events that occur during<br><br>exercise	In first two sessions, vaginal spotting (n=6), vaginal discomfort while using intra-vaginal biofeedback (n=21)	In first two sessions, vaginal discomfort (n=5)
**12**	Describe the setting in which the exercises are performed	Individual treatment room at the research center in Montreal and Sherbrooke	Pelvic floor exercise gym at the research center in Montreal and Sherbrooke
**13**	Detailed description of exercise intervention e.g. reps, sets, sessions	See eTable 2 for all the details	See eTable 2 for all the details
**14a**	Describe whether the exercises are generic (one size fits all) or tailored	Generic	Generic
**14b**	Detailed description of how exercises are tailored to the individual	The exercises were not tailored to the individual	The exercises were not tailored to the individual

**15**	Describe the decision rule for determining the starting level e.g. beginner, intermediate, advanced etc.	The starting level was standardized. All participants started at the same level, which progressed every 4 weeks<br>If the participant had difficulty completing the program, she was encouraged to do as much as possible every time.	The starting level was standardized. All participants started at the same level, which progressed every 4 weeks<br>If the participant had difficulty to complete the program, she was encouraged to do as much as possible every time.
**16a**	Describe how adherence or fidelity to the intervention is assessed/measured	The fidelity to the intervention was assessed using the written treatment protocol checklist	The fidelity to the intervention was assessed using the written treatment protocol checklist
**16b**	Describe the extent to which the intervention was delivered as planned	The intervention was delivered as planned	The intervention was delivered as planned

eTable 2. Supervised pelvic floor muscle training program

	Warm up exercises (5 minutes)	PFMT program[d] (25 minutes)				General exercises (given between PFM exercise sets) (5-10 minutes)	PFM functional training with dancing exergame (10 minutes)
		Knack[a,b]	Maximal contractions[a,b]	Fast contractions[b]	Podiums[b,c]		
**Sessions 1 to 4**	Breathing exercises (5 repetitions), anterior and posterior pelvic tilts standing (4 repetitions/2 sets), ankle flexion and extension (4 repetitions bilateral/2 sets)	(contraction + 1 cough) 3s rest 3 repetitions 3 sets	6 repetitions, 6s hold/6s rest 3 sets	6 contractions 1s hold/1s rest 3 sets	6s/6s/6s step 18s rest 3 sets	Pull belly button towards spine four-point kneeling (6 repetitions, 6s hold), bridge (6 repetitions, 6s hold)	3 beginner songs (alternate dance steps and PFM contraction)
**Sessions 5 to 8**	Breathing exercises (5 repetitions), simple rotation of the pelvis standing (4 repetitions/2 sets), ankle flexion and extension (4 repetitions bilateral/2 sets)	(contraction + 2 coughs) 3s rest 3 repetitions 3 sets	8 repetitions, 8s hold/8s rest 3 sets	8 contractions 1s hold/1s rest 3 sets	8s/8s/8s step 24s rest 3 sets	Pull belly button towards spine four-point kneeling + contraction of the pelvic floor muscle + lifting of the arm (8 repetitions, 8s hold) bridge (8 repetitions, 8s hold)	3 intermediate songs (simultaneous 1-leg dance steps and PFM contraction)
**Sessions 9 to 12**	Breathing exercises (5 repetitions)	(contraction + coughs) 3s rest 3 repetitions 3 sets	10 repetitions, 10s hold/10 rest 3 sets	10 contractions 1s hold/1s rest 3 sets	10s/10s/10s step 30s rest 3 sets	Pull belly button towards spine four-point kneeling + contraction of	3 advanced songs (simultaneous 2-leg dance steps and

	"8 shaped" rotation of the pelvis standing (4 repetitions/2 sets),<br>ankle flexion and extension (4 repetitions bilateral/2 sets)					the pelvic floor muscle + lifting of one arm and opposite leg (8 repetitions, 8s hold), bridge (10 repetitions, 10s hold)	PFM contraction)
**Week 13 to 1-year follow-up**<br>**(for home exercise only)**		(contraction + 3 coughs) 3s rest 3 repetitions 3 sets	12 repetitions, 12s hold/10 rest 3 sets	12 contractions 1s hold/1sec 3 sets	3 repetitions, 12s/12s/12s step 30s rest		

Abbreviations: PFMT, pelvic floor muscle training; PFM, pelvic floor muscles. [a] For the individual PFMT arm, the PFMT program was done with a biofeedback probe depending on the week for an average duration of 10-15 minutes per session. [b] Done through different positions depending on the session (lying, sitting, standing, standing legs open) with increasingly more time in sitting and standing. [c] Podium exercise (moderate-maximal-moderate PFM contraction): can be inverted depending on the week. [d] The PFMT program in the treatment session is used as the home PFM exercise program.

eTable 3. Baseline characteristics of the population that completed the study *vs* the population that did not complete the study

	Completed one-y follow-up (n=319)	Abandon (n=43)	*P* Value
Age, mean (SD), y	68.2 (5.9)	65.8 (4.3)	0.002
BMI, mean (SD), kg/m²	27.1 (4.6)	27.3 (3.9)	0.805
Parity, median (IQR)	2 (1-3)	2 (1-2)	0.424
Caesarean section	0 (0-0)	0 (0-0)	0.588
Vaginal delivery	2 (0-3)	2 (1-2)	0.445
Type of UI, No. (%)			0.875
Stress UI	55 (17%)	7 (16%)	
Mixed UI	264 (83%)	36 (84%)	
Duration of symptoms, mean (SD), y	9.6 (9.6[a]; n=313)	10.5 (11.5)	0.559
Number of comorbidities, mean (SD), /28[b]	3.5 (2.0)	3.3 (2.1)	0.529
Number of medications, mean (SD)	3.1 (2.1)	2.9 (2.4)	0.618
MMSE, mean (SD), /30[c]	29.0 (1.1[b]; n=317)	29.2 (0.9)	0.214
Previous surgery for UI, No. (%)	8 (3%)	3 (7%)	0.109
Current smoker, No. (%)	5 (2%[b]; n=318)	2 (5%)	0.360

Abbreviations: n, number of patients for whom data were available; SD, standard deviation; IQR, interquartile range; BMI, body-mass index, calculated as weight in kilograms divided by height in meters squared; No., number; UI, urinary incontinence; MMSE, Mini Mental State Examination. None of the between-group comparisons were significant at baseline. [a] Because of missing data, numbers do not sum to group totals. [b] Refers to the total number of comorbidities per participant as reported from a standardized list of 28 diseases and conditions: high blood pressure, osteoporosis, hypercholesterolemia, heart disease, stroke, lung disease/asthma, tuberculosis, depression, loss of vision, vascular diseases, renal failure, glaucoma, transplant, gout, fractured hip, breast cancer, rheumatoid arthritis, other arthritis (i.e., osteoarthritis), diabetes, deterioration in hearing, epilepsy, migraines, Parkinson disease, HIV, hepatic impairment, stomach ulcers, thyroid disease and colitis. [c] MMSE scores range from 0 to 30 with lower scores (17 or lower) indicating severe cognitive impairment; a score of 24 or higher indicates no cognitive impairment.

eTable 4. Mean values for the primary and urinary incontinence specific secondary outcomes in individual and group-based pelvic floor muscle training at 12-weeks and one-year follow-up[a]

	N total (Individual/ Group-based)	Individual PFMT	Group-based PFMT	Mean difference (95% CI)	*P* Value
**Leakage reduction**					
At 1-year follow-up	316 (164/152)	56% (56%)	56% (58%)	-1 (-13 to 12)	0.54
At 1-year follow-up (ITT)	362 (184/178)	50% (57%)	50% (63%)	0 (-12 to 12)	0.51
**Leakage episodes, episodes per day**					
Baseline	360 (183/177)	2.08 (1.72)	2.09 (2.41)		
After 12-week treatment	336 (171/165)	0.76 (1.01)	0.77 (0.9)	-0.01 (-0.22 to 0.19)	0.46
At 1-year follow-up	318 (165/153)	0.85 (1.18)	0.72 (0.88)	0.14 (-0.09 to 0.37)	0.88
At 1-year follow-up (ITT)	360 (183/177)	0.91 (1.18)	0.9 (1.4)	0.01 (-0.26 to 0.28)	0.53
**Urine loss on Pad test, grams per 24h**					
Baseline	351 (180/171)	21.16 (43.12)	17.25 (34.06)		
After 12-week treatment	319 (166/153)	6.44 (12.59)	6.47 (12.44)	-0.03 (-2.79 to 2.73)	0.49
At 1-year follow-up	273 (142/131)	8.49 (18.78)	4.9 (9.02)	3.6 (0.12 to 7.07)	0.98
At 1-year follow-up (ITT)	358 (183/175)	8.32 (17.65)	7.16 (19.5)	1.15 (-2.72 to 5.03)	0.72
**ICIQ-UI SF, 0-21[b]**					
Baseline	361 (184/177)	12.27 (3.12)	12.22 (3.38)		
After 12-week treatment	335 (171/164)	6.37 (3.73)	7.28 (3.89)	-0.91 (-1.73 to -0.09)	0.01
At 1-year follow-up	314 (162/152)	7.43 (4.15)	7.05 (4.49)	0.39 (-0.58 to 1.35)	0.78
At 1-year follow-up (ITT)	362 (184/178)	7.78 (4.36)	7.45 (4.72)	0.33 (-0.61 to 1.27)	0.75
**ICIQ-LUTSqol, 19-76[c]**					
Baseline	358 (182/176)	33.9 (8.59)	34.6 (9.81)		
After 12-week treatment	336 (171/165)	24.77 (5.98)	25.2 (6.08)	-0.43 (-1.73 to 0.86)	0.26
At 1-year follow-up	314 (163/151)	25.13 (7.04)	25.19 (6.89)	-0.05 (-1.6 to 1.5)	0.47
At 1-year follow-up (ITT)	361 (184/177)	25.61 (7.14)	26 (7.75)	-0.39 (-1.93 to 1.16)	0.31

Abbreviations: PFMT, pelvic floor muscle training; CI, confidence interval; ITT, intention-to-treat; ICIQ-UI SF, International Consultation on Incontinence Modular Questionnaire - Urinary Incontinence Short Form; ICIQ-LUTSqol, International Consultation on Incontinence Modular Questionnaire - Lower Urinary Tract Symptoms Quality of Life. [a] Data are expressed as mean (SD). [b] Greater values indicate an increased severity (minimal clinically important difference, 2.5 points).[1] [c] Greater values indicate an increased impact on quality of life (minimal clinically important difference, 3.7 points).[1]

eTable 5. Mean values for the impact on other lower urinary tract symptoms, vaginal, sexual symptoms and self-efficacy in individual and group-based pelvic floor muscle training at 12-weeks and one-year follow-up

	N total (Individual/Group-based)	Individual PFMT	Group-based PFMT	Mean difference (95% CI)	*P* Value
**Other LUT symptoms**					
**Micturition, mean (SD), 7-day bladder diary**					
Baseline	359 (183/176)	8.79 (3.38)	9.16 (3.66)		
After 12-week treatment	336 (171/165)	7.27 (2.76)	7.1 (2.24)	0.17 (-0.37 to 0.71)	0.73
At 1-year follow-up	318 (165/153)	7.66 (2.38)	7.4 (2.35)	0.26 (-0.26 to 0.78)	0.84
At 1-year follow-up (ITT)	360 (183/177)	7.58 (2.35)	7.38 (2.39)	0.2 (-0.29 to 0.69)	0.79
**ICIQ-N, mean (SD), 0-8[a]**					
Baseline	361 (184/177)	2.58 (1.77)	2.92 (1.76)		
After 12-week treatment	336 (170/166)	1.55 (1.37)	1.68 (1.33)	-0.13 (-0.42 to 0.16)	0.18
At 1-year follow-up	317 (164/153)	1.87 (1.52)	1.9 (1.46)	-0.02 (-0.35 to 0.31)	0.44
At 1-year follow-up (ITT)	362 (184/178)	1.85 (1.51)	1.94 (1.49)	-0.09 (-0.4 to 0.22)	0.28
**Vaginal and sexual symptoms**					
**ICIQ-VS vaginal symptoms subscale, mean (SD), 0-53[a]**					
Baseline	358 (183/175)	5.51 (5.5)	6.18 (6.18)		
After 12-week treatment	335 (170/165)	3.94 (4.57)	3.5 (4.34)	0.44 (-0.52 to 1.4)	0.82
At 1-year follow-up	315 (163/152)	3.33 (4.11)	3.22 (5.04)	0.11 (-0.92 to 1.13)	0.58
At 1-year follow-up (ITT)	362 (184/178)	3.45 (4.18)	3.72 (5.27)	-0.27 (-1.26 to 0.71)	0.29
**ICIQ-VS sexual matters subscale, mean (SD), 0-58[a]**					
Baseline	127 (70/57)	11.97 (17.51)	17.86 (21.84)		
After 12-week treatment	120 (63/57)	6.9 (13.81)	12.35 (17.62)	-5.45(-11.22 to 0.33)	0.03
At 1-year follow-up	103 (58/45)	9.45 (16.66)	7.24 (12.86)	2.2 (-3.57 to 7.97)	0.77
At 1-year follow-up (ITT)	160 (87/73)	10.15 (17.37)	11.01 (16.79)	-0.86 (-6.21 to 4.48)	0.37

**ICIQ-VS quality of life subscale, mean (SD), 0-10[a]**					
Baseline	361 (184/177)	1.85 (2.94)	2.05 (2.96)		
After 12-week treatment	334 (169/165)	0.84 (1.92)	1.01 (2.22)	-0.17 (-0.61 to 0.28)	0.23
At 1-year follow-up	314 (163/151)	0.77 (1.93)	0.65 (1.71)	0.12 (-0.29 to 0.52)	0.72
At 1-year follow-up (ITT)	362 (184/178)	0.74 (1.88)	0.87 (2.07)	-0.13 (-0.54 to 0.28)	0.27
**ICIQ-FLUTSsex, mean (SD), 0-14[a]**					
Baseline	347 (176/171)	5.86 (3.59)	6.32 (3.5)		
After 12-week treatment	325 (161/164)	5.28 (3.79)	5.42 (3.74)	-0.14 (-0.96 to 0.68)	0.37
At 1-year follow-up	309 (161/148)	5.58 (3.62)	5.94 (3.54)	-0.36 (-1.16 to 0.45)	0.19
At 1-year follow-up (ITT)	360 (184/176)	5.6 (3.64)	5.84 (3.6)	-0.24 (-0.99 to 0.51)	0.26
**Self-efficacy**					
**Geriatric self-efficacy index, mean (SD), 0-120[b]**					
Baseline	362 (184/178)	55.18 (20.75)	58.75 (22.01)		
After 12-week treatment	335 (170/165)	90.51 (18.89)	91.48 (20.29)	-0.97 (-5.19 to 3.24)	0.67
At 1-year follow-up	309 (159/150)	84.66 (22.45)	89.02 (23.37)	-4.36 (-9.49 to 0.78)	0.95
At 1-year follow-up (ITT)	362 (184/178)	83.74 (23.58)	86.75 (25.47)	-3.01 (-8.09 to 2.07)	0.88
**Adherence to home exercises, No. (%)**					
After 12-week treatment 4 to 5 times per week	(171/166)	152 (89)	142 (86)	3% (-4% to 10%)	0.36
At 1-year follow-up 3 times per week	(165/154)	48 (29)	38 (25)	4% (-6% to 14%)	0.36
At 1-year follow-up at least once per week	(165/154)	110 (67)	107 (69)	-3% (-13% to 7%)	0.63

Abbreviations: PFMT, pelvic floor muscle training; CI, confidence interval; LUT, lower urinary tract; ITT, intention-to-treat; ICIQ-N, International Consultation on Incontinence Questionnaire Nocturia Module; ICIQ-VS, International Consultation on Incontinence Questionnaire Vaginal Symptoms Module and its subscale on vaginal symptoms, sexual matters and impact on quality of life; ICIQ-FLUTSsex, International Consultation on Incontinence Questionnaire Female Sexual Matters Associated with Lower Urinary Tract Symptoms Module. [a] For all ICIQ modules, higher values indicate increased symptom severity. [b] Higher scores indicating higher self-efficacy.

eTable 6. Primary outcome- percentage reduction of leakage episodes in individual and group-based interventions at one-year follow-up according to subgroups of interest

	Individual PFMT		Group-based PFMT	
	**Median (IQR)**	**Mean (SD)**	**Median (IQR)**	**Mean (SD)**
**Centre**				
Montreal	69 (35-89)	53 (62)	72 (41-85)	50 (67)
Sherbrooke	71 (44-88)	61 (40)	75 (50-95)	66 (34)
**UI type**				
Stress UI	50 (0-71)	30 (89)	70 (52-93)	63 (38)
Mixed UI	71 (50-89)	60 (48)	74 (46-86)	54 (62)
**UI severity**[a]				
Moderate	71 (50-88)	54 (66)	75 (57-85)	56 (66)
Severe	67 (34-89)	57 (44)	69 (46-86)	58 (46)
**Age, y**				
≤70	71 (45-89)	58 (56)	73 (47-86)	58 (45)
>70	67 (32-88)	50 (58)	75 (46-91)	53 (76)
**BMI, kg/m**^{2}[b]				
Normal	71 (50-89)	56 (62)	75 (48-87)	57 (62)
High	63 (34-88)	56 (37)	66 (36-84)	57 (39)

Abbreviations: PFMT, pelvic floor muscle training; UI, urinary incontinence; IQR, interquartile range; SD, standard deviation; y, years; BMI, Body-mass index. [a] UI severity was based on the International Consultation on Incontinence Modular Questionnaire - Urinary Incontinence Short Form (ICIQ-UI SF). Scores higher than 12 were considered severe.[2] [b] The body-mass index is the weight in kilograms divided by the square of the height in meters. Body-mass indexes higher than 25 kg/m^2 were considered high.[3]

eTable 7. Odds ratios for the association between the intervention arm (group-based pelvic floor muscle training» *vs* «individual pelvic floor muscle training) and the binary outcome of percentage reduction of leakage episodes (≥50% or <50%) (1) for a full model for all participants without interaction, (2) for each stratified model of interest, and (3) for each model with interactions

	At 1-year follow-up	
	**OR (95% CI); *P* Value**	**(3) Interaction *P* Value[a]**
**(1) Full model for all participants without interaction**	1.27 (0.77 to 2.10); 0.36	
**(2) Stratified models of interest[b]**		
**Centre**		
Montreal	1.24 (0.68 to 2.30); 0.48	0.90
Sherbrooke	1.23 (0.47 to 3.26); 0.68	
**UI type**		
Stress UI	5.24 (1.40 to 23.25); 0.02	0.03
Mixed UI	0.94 (0.54 to 1.65); 0.84	
**UI severity[c]**		
Moderate	1.21 (0.58 to 2.58); 0.61	0.81
Severe	1.52 (0.74 to 3.15); 0.25	
**Age, y**		
≤70	1.20 (0.64 to 2.26); 0.58	0.75
>70	1.18 (0.48 to 2.87); 0.72	
**BMI, kg/m[2d]**		
Normal	1.02 (0.56 to 1.86); 0.95	0.16
High	2.20 (0.78 to 6.55); 0.15	

Abbreviations: OR, odds ratio; CI, confidence interval; UI, urinary incontinence; y, years; BMI, body-mass index, calculated as weight in kilograms divided by height in meters squared. [a]Given that 10 interactions are being tested, the Bonferroni correction should be used for the significance level – $\alpha_{corrected}$ = 0.005. [b]The stratification covariates considered in the different models are: center, urinary incontinence type, severity, age and body mass index. All the models are also adjusted for the following additional covariates: number of leakages at baseline and home exercise adherence. [c]UI severity was based on the International Consultation on Incontinence Modular Questionnaire - Urinary Incontinence Short Form (ICIQ-UI SF). Scores higher than 12 were considered severe.[2] [d]The body-mass index is the weight in kilograms divided by the square of the height in meters. Body-mass indexes higher than 25 kg/m^2 were considered high.[3]

eTable 8. Minimal clinically important differences at the one-year follow-up

	N total (Individual/ Group-based)	Individual physiotherapy	Group-based physiotherapy	*P* Value[a]
		No. of participants (%)		
**Leakage episodes (7-day bladder diary)[b]**				
50% reduction	318 (165/153)	118 (72%)	113 (74%)	0.64
70% reduction		83 (50%)	83 (55%)	0.48
90% reduction		37 (22%)	33 (22%)	0.85
**Urine loss (gr. on 24h pad test)[c]**				
50% reduction	161 (84/77)	54 (64%)	57 (74%)	0.18
70% reduction		42 (50%)	43 (56%)	0.46
90% reduction		16 (19%)	20 (26%)	0.29
**UI-symptoms severity[d]**				
minimum important difference	313 (162/151)	116 (72%)	110 (73%)	0.81
incremental improvement		81 (50%)	85 (56%)	0.27
**UI-specific quality of life[e]**				
minimum important difference	312 (161/151)	119 (74%)	110 (73%)	0.83
incremental improvement		90 (56%)	83 (55%)	0.87

Abbreviations: UI, urinary incontinence. [a] *P* Values were calculated with the use of the chi-square test. [b] Participants considered to recognize minimal clinically important differences at reductions of approximately 50%.[4] [c] For those with urine loss >4 gr at baseline. [d] UI-symptoms severity based on the International Consultation on Incontinence Modular Questionnaire - Urinary Incontinence. Minimal clinically important differences, 2.52 points; incremental improvement, 4.62 points.[1] [e] UI-specific quality of life based on the International Consultation on Incontinence Modular Questionnaire - Lower Urinary Tract Symptoms Quality of Life. Minimal clinically important differences, 3.71 points; incremental improvement, 6.63 points.[1]

eTable 9. Other treatments taken during the study period

	Individual PFMT (n=184)	Group-based PFMT (n=178)
**Visits to health professionals**		
Acupuncturist	2 sessions (n=1)	
Osteopath		1 session (n=1)
Physiotherapist		3 sessions (n=1)
**Medication for urinary urgency**		
Oxybutynin	daily for 3 days (n=1)	daily for 1 month (n=1)
Solifenacin		daily for 6 months (n=1); daily for 1 week (n=1)
Trospium	daily for 8 months (n=1)	
**Other**		
Medication unknown from RCT	daily for 1 month (n=1)	
Estradiol		11 months (n=1)
Nitrofurantoin		daily for 1 week (n=1)
Herbal tea	daily for 6 months (n=1)	
**Total**	5 (3%)	7 (4%)

Abbreviations: PFMT, pelvic floor muscle training; n, number of patients for whom data were available; RCT, randomized control trial.

eReferences

1. Nyström E, Sjöström M, Stenlund H, Samuelsson E. ICIQ symptom and quality of life instruments measure clinically relevant improvements in women with stress urinary incontinence. *Neurourol Urodyn*. 2015;34(8):747-751. doi:10.1002/nau.22657
2. Klovning A, Avery K, Sandvik H, Hunskaar S. Comparison of two questionnaires for assessing the severity of urinary incontinence: The ICIQ-UI SF versus the incontinence Severity Index. *Neurourol Urodyn*. 2009;28(5):411-415. doi:10.1002/nau.20674
3. WHO - Regional office for Europe. Body mass index - BMI. Health Topics. http://www.euro.who.int/en/health-topics/disease-prevention/nutrition/a-healthy-lifestyle/body-mass-index-bmi. Accessed February 24, 2019.
4. Yalcin I, Peng G, Viktrup L, Bump RC. Reductions in stress urinary incontinence episodes: what is clinically important for women? *Neurourol Urodyn*. 2010;29(3):344-347. doi:10.1002/nau.20744
