# Supplementary material for: Group-Based vs Individual Pelvic Floor Muscle Training to Treat Urinary Incontinence in Older Women: A Randomized Clinical Trial
Source: JAMA Intern Med. 2020 Aug 3;180(10):1284–93. doi: 10.1001/jamainternmed.2020.2993 (PMC7400216; doi:10.1001/jamainternmed.2020.2993)
Supplement: Supplement 3. — Data Sharing Statement [file jamainternmed-e202993-s003.pdf]

Data Sharing Statement

Data
Data available: Yes
Data types: Deidentified participant data
How to access data: data will be available on request to Prof Dumoulin. Email: chantal.dumoulin@umontreal.ca
When available: beginning date: 05-01-2021, end date: 05-01-2026

Supporting Documents
Document types: None

Additional Information
Who can access the data: researchers whose proposed use of the data has been approved by ethics committee
Types of analyses: for any purpose
Mechanisms of data availability: with investigator support
